# Applying multi-omics techniques to the discovery of biomarkers for acute aortic dissection

**DOI:** 10.3389/fcvm.2022.961991

**Published:** 2022-12-15

**Authors:** Xinyu Hao, Shuai Cheng, Bo Jiang, Shijie Xin

**Affiliations:** ^1^Department of Vascular Surgery, The First Affiliated Hospital of China Medical University, China Medical University, Shenyang, China; ^2^Key Laboratory of Pathogenesis, Prevention and Therapeutics of Aortic Aneurysm, Shenyang, Liaoning, China

**Keywords:** multi-omics, acute aortic dissection, biomarkers, high-throughput sequencing technology, mass spectrometry technology, diagnostics, integrated strategies

## Abstract

Acute aortic dissection (AAD) is a cardiovascular disease that manifests suddenly and fatally. Due to the lack of specific early symptoms, many patients with AAD are often overlooked or misdiagnosed, which is undoubtedly catastrophic for patients. The particular pathogenic mechanism of AAD is yet unknown, which makes clinical pharmacological therapy extremely difficult. Therefore, it is necessary and crucial to find and employ unique biomarkers for Acute aortic dissection (AAD) as soon as possible in clinical practice and research. This will aid in the early detection of AAD and give clear guidelines for the creation of focused treatment agents. This goal has been made attainable over the past 20 years by the quick advancement of omics technologies and the development of high-throughput tissue specimen biomarker screening. The primary histology data support and add to one another to create a more thorough and three-dimensional picture of the disease. Based on the introduction of the main histology technologies, in this review, we summarize the current situation and most recent developments in the application of multi-omics technologies to AAD biomarker discovery and emphasize the significance of concentrating on integration concepts for integrating multi-omics data. In this context, we seek to offer fresh concepts and recommendations for fundamental investigation, perspective innovation, and therapeutic development in AAD.

## 1 Introduction

### 1.1 Applying multi-omics concepts to biomedicine

**FIGURE 1:**
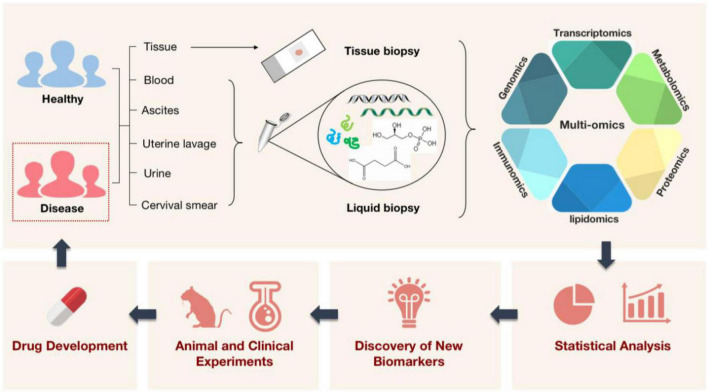
A schematic illustration of how multi-omics technologies are employed in biomedicine. Multi-omics technologies are used to process tissue samples from people, animals, and the environment for high-throughput quantitative analysis of molecules in biological systems. This aims to reveal disease-related etiological mechanisms, find new diagnostic markers, shed light on disease pathogenesis, and pave the way for the creation and application of new targeted therapeutic agents with the ultimate goal of treating diseased populations.

Biomedicine is an important engineering field related to the improvement of medical diagnosis and human’s own health. As science has advanced over the past few decades, it has become evident that many diseases, particularly those that are challenging to diagnose and cure, are frequently brought on by a confluence of genetic and environmental variables (1). When the idea of “omics” was first introduced, a new era had begun in which people were no longer constrained to using solitary factors to explain illness. This meant that we started to evaluate a set of molecules in a comprehensive way (2). One by one, the traditional ideas of genomics, transcriptomics, proteomics (3), and metabolomics were introduced. Single-omics methods, however, are only able to explain diseases at a single level (4) (e.g., at the gene, RNA, or protein level) and are unable to fully depict the complex biological processes involved (5), especially the regulatory and signaling mechanisms (6). In light of this, scientists’ interest in the integration of several histology techniques has increased recently. It is a method that has the potential to broaden our understanding of biology and disease (1) as well as the diversity and depth of the biological sciences (7). The molecular underpinnings of complex diseases like cancer (8–10), immune system diseases (11, 12), infectious diseases (1, 13), and cardiovascular diseases (14) can be better understood by the analysis of multi-omics data. Thus, we can reveal disease-related etiological mechanisms, explore new biomarkers, and develop new targeted drugs for clinical research applications. To advance the development of clinical precision medicine (15) and bring benefits to patients (Figure 1).

### 1.2 Advantages and prospects of applying multi-omics concepts in AAD

**FIGURE 2:**
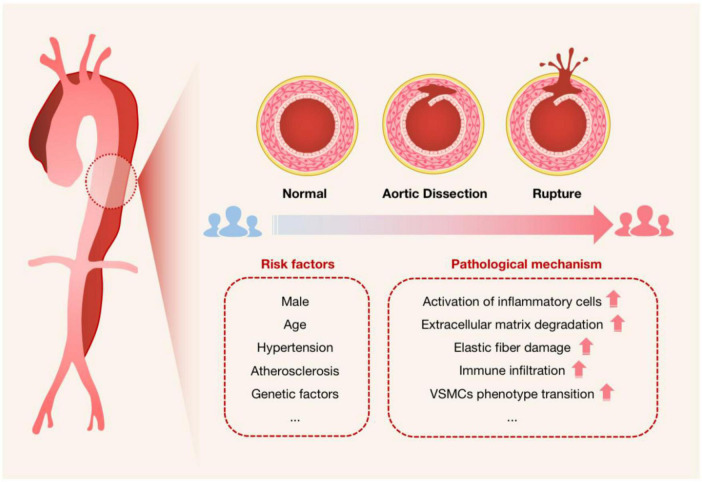
Schematic diagram used to characterize the onset of AAD. The cross-sectional schematics of a normal artery, an artery with aortic dissection, and an artery with dissection rupture are shown. It is intended to show the progression of AAD pathogenesis, i.e., the blood flow breaks through the intima of the aorta and enters the tunica media, forming a false lumen that will be at risk of rupture if not treated promptly, especially for Stanford type A dissection. Risk factors for AAD and possible associated pathological mechanisms are also listed.

Acute aortic dissection (AAD) is a cardiovascular disease with a high mortality risk, in which blood flow breaks through the intima of the aorta and enters the tunica media, forming a false lumen. The true lumen and the false lumen may or may not be connected, and blood flowing between them may form a thrombus. There has been an upsurge in AAD cases recently (16), with a trend toward younger ages (17). Aging, high blood pressure, atherosclerosis, and connective tissue-related genetic risk factors are among the many risk factors linked to AAD (18). Additionally, men are much more likely to experience it than women are (19) (Figure 2). AAD can be divided into acute type A dissection and acute type B dissection according to the anatomical location of the lesion. Acute type A dissection involves the ascending aorta and is treated clinically with emergency open surgery such as aortic replacement, while acute type B dissection involves only the thoracic descending aorta and its distal parts and is treated mainly with drugs or intervention (17). Clinically, patients with AAD tend to present with tearing-like pain, and the main site of pain correlates with the site of dissection, with chest pain being the most common. However, because the symptoms are not diagnostically specific, they are easily confused with other diseases such as acute coronary syndrome (ACS), acute myocardial infarction (AMI), and acute abdomen. And in about 6% of cases, no symptoms of chest pain are present (20), making AAD often misdiagnosed or missed (21). AAD rupture can have serious side effects, including extensive bleeding that quickly results in inadequate perfusion of several organs and eventual death (22). In patients with untreated symptoms caused by overlook or misdiagnosis, the mortality rate can range from 1 to 2% per hour following the beginning of symptoms to 50% at 48 h (19, 21).

Currently, the most common method used in hospitals for the diagnosis of AAD is laboratory tests combined with imaging (23). In order to further reduce the possibility of underdiagnosis and misdiagnosis, the use of omics techniques becomes an inevitable choice to drive technological innovation when we try to optimize the existing diagnostic protocols to discover new, biologically based, low-budget, easily accessible, highly sensitive, specific and non-invasive prognostic and diagnostic tools. We have gradually connected molecule-to-molecule and molecule-to-phenotype linkages in organisms by merging multi-omics, starting at the genetic level and moving up via nucleic acids, proteins, and tiny molecules of metabolism. At the same time, AAD, as a highly dynamic and changing disease, produces largely different specific biological indicators at each stage of its development. To develop a thorough biological model, more attention must be paid to longitudinal molecular alterations (i.e., various molecular layers) in the same or the same group of individuals (24). It also leads to a deeper comprehension of how signaling pathways and network interactions affect disease pathogenesis mechanisms (25). Additionally, these comprehensive data reveal several pathological features of AAD, which is crucial for patient risk classification and optimizing therapies to stop AAD progression (14).

## 2 Introduction of omics techniques

### 2.1 Genomics and transcriptomics

**FIGURE 3:**
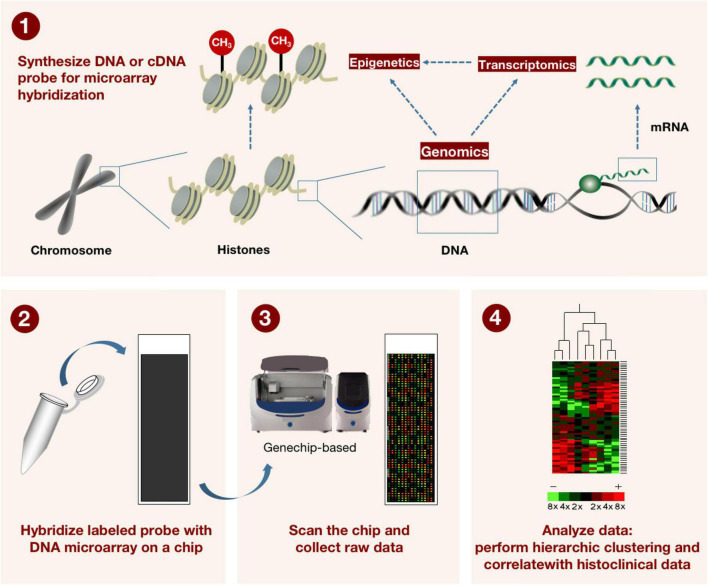
The method for describing how microarray technology is applied in genomics and transcriptomics. DNA or cDNA probes for microarray hybridization are first synthesized, and the labeled probes are subsequently hybridized to the DNA microarray on the microarray. The microarray is scanned by machine and raw data is collected. Finally, a hierarchical grouping and correlation with tissue clinical data was performed on the data. This in turn can be used to detect known gene sequences with high throughput.

Since its inception more than ten years ago, genome-wide association analysis (GWAS) has significantly advanced our understanding of human susceptibility genes and their roles in common diseases. GWAS attempts to find locus variants associated with complex features in populations, and in particular to detect connections between common single nucleotide polymorphisms (SNPs) and a wide range of prevalent diseases (26). It inaugurated a new era in our understanding of the genetic origins of disease. Additionally, DNA microarrays (also known as genotyping microarrays) with known high-frequency SNPs are frequently used in such research (Figure 3). The number of variants that can be detected using genotyping microarrays has increased over the years, but even the high-density 5 million SNP microarray (Illumina OMNI5) (27) covers only a small fraction of the 3.3 billion bases in the human genome. Additionally, some SNPs with low frequency are frequently deleted after applying conventional quality control (QC) before association analysis, despite the fact that they could be crucial to understanding some rare diseases or phenotypes (28).

Following GWAS, sequencing technologies reached a historical turning point. Due to their reduced cost, broader detection range, and improved ability to discover uncommon genetic variants (e.g., point mutations, copy number variants, and structural variants) (29), high-throughput whole genome sequencing (WGS), whole exome sequencing (WES), and simplified genome sequencing (RRGS) are anticipated to displace microarray-based genotyping approaches in GWAS. In particular, WES technology allows better detection of pathogenic mutations present in coding regions or typical splice sites, allowing the study of up to 85% of Mendelian diseases (30). In the history of sequencing technology, second-generation sequencing (NGS) has been redefined in large part by improvements in microfabrication and high-resolution imaging that allow us to perform a large number of parallel sequencing responses at the micron scale (31). The division between NGS and third-generation sequencing (TGS) technologies is based on the development of real-time single-molecule sequencing (SMS) and nanopore sequencing (32). SMS makes it possible to directly sequence individual DNA molecules, doing away with the requirement for PCR amplification stages and cutting down on amplification-related mistakes. While this is happening, nanopore sequencing technology uses its own high accuracy, longer read length, and higher throughput of DNA or RNA molecules to apply to professional fields like genome assembly, full-length transcript detection, base modification detection, clinical diagnosis, and epidemic monitoring, greatly improving the quality and reliability of data (33).

If genomics is the study of the sequence and structural loci of the genes themselves, transcriptomics explores the function and structure of genes in an organism by looking at the level of gene expression regulation in turn. Transcriptome analysis focuses on gene expression levels and is crucial for understanding the structure and function of the genome, unraveling the genetic networks underlying disease, and discovering molecular biomarkers responsive to infections, medications, and disease states (34). RNA-seq technology enables more efficient, less expensive quantification of gene transcript levels. Most importantly, the discovery of novel genes and RNAs opens up new opportunities for the molecular biology sector to address clinical medical issues (35). Single cell RNA sequencing (scRNA-seq) enables transcriptome-wide analysis of individual cells, which can be used to study cell subpopulations (36) and uncommon cell types within tissues (37), involves isolating and lysing the individual cells, converting their RNA to cDNA, and amplifying the cDNA to produce high-throughput sequencing libraries (38).

By putting tissue sections on arrays of retrotransposon primers with specially placed barcodes, spatial transcriptomics enables the viewing and quantitative analysis of the transcriptome at spatial resolution in a single tissue section (39). It can replace the vacuum left by high-throughput techniques, which, since they cannot be used *in situ*, lose information about the spatial relationships between cataloged cell populations, by giving us unbiased maps of spatial composition for creating tissue atlases (40). Local networks of *in situ* intercellular communication can be better understood by spatial transcriptomics, and there is mounting proof that the tissue microenvironment of cells can affect their phenotypic. The molecular, cellular, and geographical structure of disease ecological niches (41) can be discovered by combining scRNA-seq with regionally resolved transcriptomics, multiplex *in situ* hybridization, *in situ* sequencing, and spatial barcoding approaches to localize RNA inside tissues (36).

### 2.2 Proteomics and metabolomics

Proteins have an irreplaceable role in exercising biological functions, and most of the functional information in an organism can be reflected by protein expression, structure, function, interactions and modifications (42). It can be said that proteins are the most direct presentation of the biological effects of a gene. Marc Wilkins coined the term “proteome” in 1996 (3), and it offers information that is complimentary to those of genomes and transcriptomics. However, proteomes are frequently substantially more complicated than their genomic and transcriptomic equivalents, and post-translational modifications (PTMs) in particular greatly contribute to the higher diversity of protein forms (43).

In the early days of proteomics research, reducing sample analysis time while increasing the depth of proteome coverage was always the goal of scholars (44). The subsequent separation of complicated protein samples was made possible by the development of mass spectrometry (MS) and Edelman sequencing technologies. Then, isotope labeling, isobaric labeling, and metabolic labeling-based technologies called ICAT, iTRAQ (45), and SILAC (46) were introduced. The development of proteomics reached a pinnacle with the introduction of high-throughput sequencing technologies like X-ray crystallography (47), which can determine the three-dimensional structure of proteins as an anchor for structural biology, and NMR spectroscopy (48), which is used to resolve high-resolution protein structures (49). For decades, MS has been used extensively in the field of biomolecules, including high-throughput protein analyses that are both qualitative and quantitative, high-throughput analyses of protein post-translational modifications, identification of regulatory networks and protein-protein interactions, identification of protein-small molecule interactions, identification of biomarkers, and screening of potential drug targets (50). Tandem mass spectrometry combined with liquid chromatography (LC-MS/MS) has been used in a variety of settings. The fields which heavily relying on LC-MS/MS methods including clinical toxicology, clinical endocrinology, and validation of immunosuppressive medications (51).

In addition to being relevant to proteins and peptides, MS technique is crucial for the investigation of metabolic small molecules. The term “metabolome” was originally used by Oliver et al. in 1998 (52), and its analysis offers a novel method for the study of cellular metabolism and overall regulation in the fields of biochemistry and molecular biology. There are several ways to research metabolomics, however, at this point, nuclear magnetic resonance (NMR) spectroscopy and mass spectrometry (MS) metabolomics analysis are the most popular and encouraged methodologies. As a separation technique, spectroscopy can separate compounds with similar structures, such as isomers, to reduce the complexity of biological extracts, and then send them to mass spectrometry for subsequent analysis, which will greatly improve the sensitivity and specificity of metabolomics analysis techniques. While metabolite culture supernatants are taken from tissues, biofluids, or cells, and then purified and injected by gas or liquid chromatography, MS is applied to metabolomics and proteomics in essentially the same ways. They are then measured for molecular mass-to-charge ratios and fragmentation patterns, which are compared to databases of known and anticipated compounds. Here, liquid chromatography-mass spectrometry (LC-MS) has been the method of choice for non-targeted metabolomics because it is the most adaptable and well-liked separation technology (53).

**FIGURE 4:**
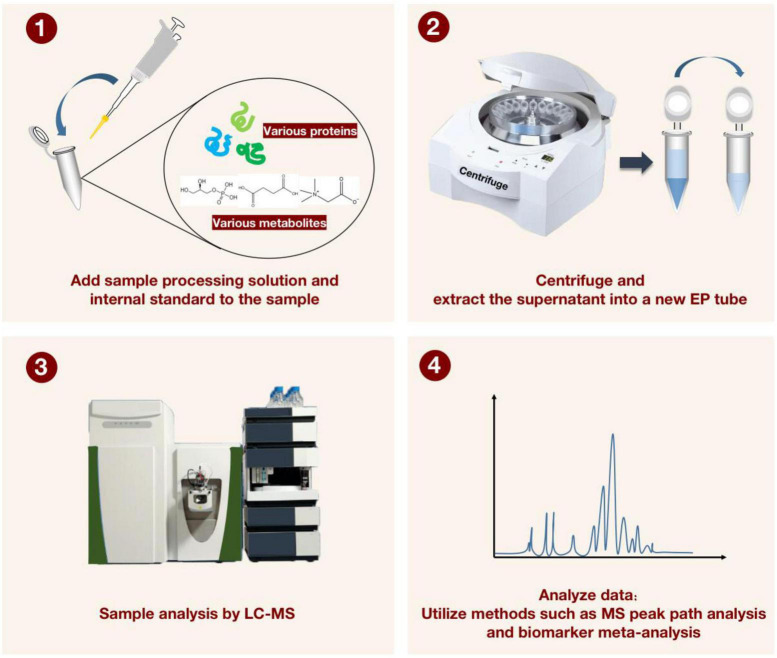
For characterizing the detection process of liquid chromatography-mass spectrometry (LC-MS) applied to proteomics and metabolomics. The sample processing solution and the internal standard solution are first added to the sample, and after vortexing and centrifugation, the supernatant is transferred to a new EP tube. The LC-MS instrument was then applied to manipulate the processed samples. Finally, MS peak path analysis and biomarker meta-analysis are performed to generate visualized data.

While the aforementioned analytical techniques are crucial, the choice of method and the development of the post-analytical data processing also significantly affect the outcomes (53). For currently tested metabolites, there are still major technical obstacles to data analysis and data integration (54). Recently, MetaboAnalyst’s knowledge base was updated to include MS peak pathways, biomarker meta-analysis, a web-based resource manager for building networks of multi-omics technologies, and many other useful tools, opening up more possibilities for the future of metabolomics to have greater reproducibility and transparency in the interpretation of data analysis (55) (Figure 4).

## 3 Discovery of AAD biomarkers based on multi-omics technologies

Biomarker profiles are defined as indicators that can be measured in body fluids and tissues for the objective assessment of normal biology, pathological processes and pharmacological responses during actual treatment (56, 57). They have been divided into three major categories: molecular (e.g., chemicals, proteins, or genes), cellular (e.g., cell kinds, cell shape, and tissue histology), and imaging (e.g., X-ray, CT, PET or MRI features) (58). And in the clinical phase, a good biomarker must serve the purpose of “improving patient prognosis”.

Most of the biomarkers for AAD at this stage have been discovered based on their possible pathological mechanisms. But regarding the pathogenic mechanisms underlying the development of AAD, there are currently a variety of conflicting and ambiguous views. Most researchers agree that two factors are primarily responsible for the pathogenesis of AAD: first, the pathological alterations in the vessel wall itself, including the activation of inflammatory cells, the breakdown of extracellular matrix, the destruction of elastic fibers, immune infiltration, and the loss and degeneration of smooth muscle cells (59), and second, the impact of mechanical shear stress on the vessel wall caused by blood flow (60), which is frequently closely related to unmanageable hypertension. Unfortunately, no diagnostic marker has been found to be as sensitive for AAD as BRCA for hereditary breast cancer or PSA for prostate cancer diagnosis and treatment. Therefore, the introduction of high-throughput histological technologies is particularly important for exploring and building a more complete network of AAD biologic diagnostic markers. In the following, we combine genomics, transcriptomics, proteomics and metabolomics with acute aortic coarctation in Pubmed, respectively, and summarize the biomarker findings in each panel, hoping to bring inspiration on the development of early diagnostic strategies for AAD.

### 3.1 Genomics and the discovery of AAD biomarkers

The risk factors for AAD contain many gene-related pathogenic mutations (61). Renard et al. (62) used the Clinical Genome Resource (ClinGen) framework to screen 11 genes from 53 candidates thought to be strongly associated with the development of hereditary thoracic aortic aneurysm and dissection (HTAAD), namely ACTA2, MYH11 [mainly involved in the smooth muscle cell contractile system (63)], MYLK, LOX and PRKG1 [aggressive in early onset (64) and associated with a reduced smooth muscle cell contractile phenotype (65)] associated with HTAAD; FBN1 associated with Marfan syndrome; COL3A1 associated with Ehlers-Danlos syndrome; and SMAD3, TGFB2, TGFBR1 and TGFBR2, which are associated with Loeys-Dietz syndrome and mainly involve components of the TGFβ signaling pathway (66). Of these, exome sequencing revealed that SMAD3 mutations cause 2% of familial thoracic aortic aneurysms and dissections (TAAD) (67). As a result of the identification of fresh missense mutations in SMAD3’s evolutionarily conserved areas, Loeys-Dietz syndrome (LDS) type 3 has also been more precisely identified (68, 69). Similarly, a nonsense variant and four missense variants in the MH2 structural domain of SMAD2 (70) and a heterozygous missense variant in the MH1 structural domain of SMAD4 were detected by sequencing in patients with sporadic thoracic aortic disease and published as first reports (71), perhaps in relation to its important role in the proliferation of vascular smooth muscle cells (VSMCs), extracellular matrix maintenance and vascular remodeling related (72).

As a result, numerous modes of variation might appear when multiple susceptibility loci exist for the same gene, raising the risk of disease and making diagnosis more challenging (73). A pathogenic missense variation [c.6661T > C, p.(Cys2221Arg)] and two truncated variants [c.4786C > T, p.(Arg1596Ter)] and [c.6366C > CA, p.(Asp2123GlufsTer5)] of the FBN1 gene were found in three patients with Stanford B aortic dissection (74). Patients with truncating and splicing mutations were more likely to develop severe aortic dissection than those with missense mutations, especially the shift mutations (82.76% vs. 42.86%) (75). Furthermore, there are also data showing that patients with AAD with FBN1 mutations develop the disease at a younger age than those without FBN1 mutations (76). There are many similar rare mutations in common pathogenic genes, with the identification of heterozygous rare variants c.839G > T (p.Ser280Arg) (77) and c.893T > G (p.Met298Arg) (78) missense mutations in LOX, encoding a lysyl oxidase, leading to the development of ascending aortic dissection. We also discovered 9 of 338 individuals with heterozygous uncommon LTBP3 variations in a previous study, including code-shifting variants, coding deletions, nonsense variants, and five missense replacements (79). Furthermore, mosaic variations have recently been found in NGS data from TAAD patients, while being frequently missed in routine molecular diagnosis (80).

In addition, the genetic heterogeneity of TAAD was confirmed by a study that identified multiple loci affected by rare copy number variants (CNVs) in one third of patients with early-onset TAAD (ETAAD) (81). The human genome contains many CNVs, which cover a lot more nucleotides than single nucleotide polymorphisms (SNPs) and dramatically increase the diversity of genetic variation seen in the genome. Though CNVs are challenging to find using standard NGS analysis, we found six CNVs using XHMM (an algorithm that hunts for CNVs in NGS data), including four intragenic (multiple) exon deletions in MYLK, TGFB2, SMAD3, and PRKG1 (82). And on the long arm of chromosome 10, one patient had a significant (>1,000,000 bp) CNV with 11 genes lost, including the full actin alpha 2 (ACTA2) gene (74). On the other hand, it has been discovered that 22 missense variants in ACTA2, which codes for α-smooth muscle actin (83), all increase the risk of AAD. Other genes, like the CDKN2A (cell cycle protein-dependent kinase inhibitor 2A) and CDKN2B genes on chromosome 9p21.3, which encode the senescence markers p16 and p15, have also been found to have genetic variations that have been linked to various vascular diseases and may contribute to aneurysm formation and aortic dissection (63).

Acute aortic dissections (AAD’s) development and progression are also impacted by altered epigenetic control of genes. In tissue from patients with thoracic aortic dissection (TAD), analysis of cell-free DNA (cfDNA) by whole genome bisulfite sequencing (WGBS) has revealed a number of differentially methylated regions (DMRs), with associated genes enriched in the vascular system and cardiac developmental regions. We also found that alterations in DNA methylation in TAD subsequently altered the expression of these genes. The expression levels of HOXA5, HOXB6 and HOXC6, for example, were significantly downregulated in TAD patients compared to healthy controls, with the expression levels of HOXA5 and HOXB6 significantly correlated with their methylation levels (84, 85). Moreover, Hox genes are known to play an important role not only in regulating cell proliferation, differentiation and migration, but also as regulators of phenotypic plasticity in VSMCs, which are closely associated with cardiovascular development and disease. The same enrichment of motifs involved in cellular component organization, enzyme-linked receptor protein signaling pathways (which may play a key role in the development of cardiopulmonary dysfunction), and revascularization was observed when MMP2, MMP14, and WNT2B genes were discovered in a different study. The MMP2 promoter’s CpG site at position 2 was considerably more methylated in the TAD group (86). The deletion of methylation at non-CpG sites in AAD is caused by increased cell proliferation, which is linked to inflammatory vascular remodeling processes and environmental risk factors like smoking (87).

As more and more data are generated from the application of genomics to AAD, it is becoming clear that these mutated genes are often associated with pathological mechanisms of AAD pathogenesis. The risk of acute aortic events is complicated by a range of genetic variants affecting the development and remodeling of the thoracic aortic wall (88), and we also prefer to consider these pathogenic variants as genetic triggers of AAD. The likelihood of these genes being mutated to cause AAD increases when they are involved in the cell cycle (89), collagen metabolism (90), extracellular matrix maintenance (91, 92), vascular remodeling, smooth muscle cell proliferation, and the onset of hypertension (93). The occurrence of all these rare variants will need to be verified in the future by testing large samples and improving the precision of our technology. More interestingly, we discovered that carriers of pathogenic variants had significantly earlier onset of aortic dissection, higher rates of root aneurysms, lower rates of hypertension and smoking, and a higher incidence of aortic disease in family members when compared to patients without genetically pathogenic variants (94). High-throughput sequencing methods have made it possible to identify differentially expressed genes in AAD as well as in the general population. This is indicative of a variety of structural changes in the aorta that are largely genetic in origin. Gene-gene and gene-environment interactions can thus be investigated in relation to the risk of AAD by employing genetic determinants of human anatomy to comprehend cardiovascular development (95). A road map is also provided by improving the prognosis of thoracic aortic disease (96) (Table 1).

**TABLE 1:** Non-exhaustive list of biomarkers for AAD elucidated by genomics-based techniques.

Biomarker	Technology	Valuable considerations	References
ACTA2, MYH11, MYLK, LOX, PRKG1, FBN1, SMAD3, TGFB2, TGFBR1, TGFBR2, COL3A1	DNA sequencing, ClinGen framework	Some genes were found to have rare mutations in genetic loci and were considered to be closely related to HTAAD.	(62, 74, 78, 82, 83)
SMAD2, SMAD4	Genome sequencing	Rare mutations in genetic loci identified in sporadic AAD.	(70, 71)
LTBP3	WES	Rare mutations in genetic loci identified in sporadic AAD.	(79)
CDKN2A, CDKN2B	GWAS	Found to be involved in the pathogenesis of AAD and other arterial diseases.	(63)
HOXA5, HOXB6, HOXC6	WGBS	DMRs in tissues from patients with TAD, associated genes in the vasculature and Heart developing regions are enriched.	(84, 85)
MMP2, MMP14, WNT2B	Methylation microarray, Bisulfite pyrosequencing	The methylation of the second CpG site of the MMP2 promoter in the TAD group was significantly increased and an enrichment of loci involved in cellular component organization, enzyme-linked receptor protein signaling, and vascular remodeling was also observed.	(86)
USP15, SVIL	TWAS	The high expression of USP15 was associated with increased ascending aortic diameter, whereas high expression of SVIL was associated with increased diameter of the descending aorta.	(117)

### 3.2 Transcriptomics and the discovery of AAD biomarkers

In recent years, transcriptomics has been applied to the study of AAD through the use of RNA-seq methods to identify differentially expressed genes (DEGs) between AAD patients and healthy or other disease populations, as well as bioinformatics methods like weighted gene correlation network analysis (WGCNA), GO functional annotation, KEGG pathway enrichment analysis, and regional variation analysis of genomic features. The data are then further mined, and a three-dimensional understanding of AAD begins to emerge.

The Hub gene screening method also aids in the quick identification of crucial genes, since hub genes are found at nodes in the gene network and typically play an important role in the onset of disease. The identification of crucial pathway targets can be aided by the prediction of hub genes using molecular complex assay (MCODE) and CytoHubber analysis, and the pathway’s influence on disease can be more clearly determined by their knockdown or editing. Currently, many transcriptomics studies have used protein-protein interaction (PPI) networks to show the top-ranked hub genes by Cytohubber calculations, and when we statistically summarized the data, we found that the screening of hub genes did not overlap well in different datasets of different studies, which may be related to the ethnicity, type of disease, individual differences and screening conditions of the affected population. This might be related to variations in the affected population’s ethnicity, disease type, individual factors, and screening conditions. Surprisingly, even though there was not much overlap between the independent genes, GO functional annotation and KEGG pathway enrichment analysis showed that these genes were primarily enriched in inflammatory cell activation, extracellular matrix degradation, endothelial cell apoptosis, vascular smooth muscle contraction, oxidative stress, adhesive plaques, protein kinase activity, adrenergic signaling, cell cycle-related, oocyte meiosis, luteinizing hormone mediated oocyte maturation and p53 signaling pathway among other related pathways (97–101).

Among them, inflammation takes place virtually constantly as AAD develops. According to earlier research, the pro-inflammatory and pro-apoptotic activity of myocardin-related transcription factor A (MRTF-A), which is induced by the hormone angiotensin II (Ang-II), aids in the development of AAD (102). FKBP11 promotes inflammation by allowing monocytes to colonize the aorta and degrade proteins (103). Activation of Janus kinase 2 (JAK2) (104, 105) and elevated plasma ANGPT2 levels (106, 107) are suggestive of upregulation of pro-inflammatory cytokines in AAD. In turn, these upregulated proinflammatory cytokines lead to activation of chemotactic and other immune cells, especially neutrophils and T cells, which can further secrete inflammatory factors and granzyme, exacerbating the medial lesion and leading to the development of aortic dissection (108). As for mesenchymal stem cells (MSC), because of their anti-inflammatory and repairing effects, whether they can be used in the treatment of AAD needs to be explored in more depth in the future. According to a specific study, RNA sequencing revealed that the genes CXCL1, CXCL5, HTR7, and SNAP25, which were upregulated, and EMX2, NCAM1, and IGFBP2, which were downregulated, were significantly differentially expressed in MSCs with aortic dissection. Adhesion-related signaling pathways were also significantly altered by enrichment analysis, indicating that MSCs treat AAD may involve adhesion function as well as anti-inflammatory mechanisms (109). Extracellular matrix degradation is also a significant pathogenic mechanism in AAD. Sequencing technology and genealogical co-isolation analysis identified 257 pathogenic or potentially pathogenic genes, accounting for 88.89% (64/72) of all genes encoding the collagen and matrix metalloproteinase systems, indicating that collagen loss and destruction as well as disruption of the matrix metalloproteinase system may be significant in the pathogenesis of AAD (90). In addition, LGMN (legumain), as a lysosomal cysteine protease, is mainly expressed in CD68-positive macrophages and has been shown to degrade extracellular matrix components directly or through activation of downstream signals. Besides, LGMN has also been shown to inhibit integrin αvβ3 by binding to it, reducing Rho GTPase activation, downregulating VSMC differentiation markers, and worsening the progression of thoracic aortic dissection (110).

Acute aortic dissection (AAD) has a reasonably diverse cellular landscape that is separated into immune and non-immune cells. Among them, immune cells include B cells, natural killer T cells, macrophages, dendritic cells, neutrophils, and mast cells; non-immune cells include endothelial cells, fibroblasts, and VSMC (111). With the help of single-cell RNA sequencing (scRNA-seq) technology, it is possible to understand the cellular composition of the aorta wall and the gene expression of specific cells. Major immune cell subset ratios have been observed to differ significantly between AAD and normal aorta tissues, with T cells, B cells, and natural killer (NK) cells ratios being higher in AAD tissue samples. Interestingly, macrophages were found to infiltrate only at the onset of entrapment, in contrast to the long-term infiltration of macrophages and chronic degradation of the extracellular matrix that can be observed in abdominal aortic aneurysms (AAA). At the same time, depletion of circulating monocytes significantly reduced the incidence of AAD, suggesting that macrophage infiltration in the aorta is more a cause than a consequence of AAD, suggesting that macrophage infiltration is more of a “triggering” event in AAD (108). The primary molecular traits of different cell types can also be further resolved, enabling higher resolution identification between different subtypes of the same cell to reveal functional differences. This can then lead to an in-depth study and interpretation of mechanisms that might be present in AAD, such as intercellular interactions (112). Another scRNA-seq-based investigation identified seven main DEGs (ACTA2, IL6, CTGF, BGN, ITGA8, THBS1, and CDH5) and significant alterations in the relative number of VSMCs. Additionally, we discovered that single cell trajectory analysis allowed VSMCs to differentiate into 8 distinct subtypes. And the up - regulation of genes associated with the synthetic phenotype, such as matrix metalloproteinase, inflammatory cytokines, and bone bridging protein, was also found in VSMCs (113), indicating that these cells underwent a transition from the contractile to the synthetic phenotype (111), with more abnormal proliferation and migration. The down-regulation of integrin α9 (ITGA9) (114) and the up-regulation of tRF-1:30-chrM.Met-CAT (115) and CDK1 (98) facilitated exactly the occurrence of the VSMCs-related changes mentioned above. At the same time, morphological changes in endothelial cells in AAD patients, such as endothelial cell hyperplasia, loose cell junctions, and endothelial cell desquamation in aortic segments, suggest that endothelial cells are damaged in AAD patients, which may likewise underlie the pathogenesis of AAD (107). Interestingly, RBBP8/NOTCH1 was found to act as a linking molecule between DNA damage/repair and extracellular matrix (ECM) tissue, and we speculate that excessive DNA damage is a characteristic pathological change in sporadic aortic dissection (116), which may be associated with massive cellular damage and degeneration.

It should be noted that genes frequently express themselves in a selective manner and are influenced by environmental, temporal, and spatial factors. Awareness tissue function and pathological alterations requires an understanding of the biological positional context of gene expression. Scientists have also been working to address the problem of average transcriptome and spatial information loss in RNA-seq. The idea of spatial transcriptomics, which enables visualization and quantitative analysis of the transcriptome at spatial resolution in individual tissue sections and allows precise localization of gene expression events to particular locations in biological tissues, was only first introduced by STAHL et al. in 2016 (39). When spatial transcriptomics techniques are applied to AAD, it is possible to analyze individual genes’ intra- and intercellular expression levels more thoroughly. These levels are influenced by the different anatomical regions of the aorta in which they are located (117) as well as by interactions between various cell types in the tissue microenvironment. This compensates for the lack of single-cell sequencing by enabling us to initially examine and visualize the heterogeneity of different anatomical parts of aorta and correlate cell type-specific gene expression to certain anatomical structural areas. By spatial transcriptomics, Li et al. measured differences between genes that were highly expressed in the Intima, media and adventitia of arteries, respectively (118). This finding may indicate that various cellular elements in the three aortic membranes each contribute in a unique way to the development of AAD (Table 2).

**TABLE 2:** Non-exhaustive list of biomarkers for AAD elucidated by transcriptomics-based techniques.

Biomarker	Technology	Valuable considerations	References
CDC20, AURKA, RFC4, MCM4, TYMS, MCM2, DLGAP5, FANCI, BIRC5, POLE2	mRNA microarrays	qRT-PCR results showed that the expression levels of all hub genes in OA samples were significantly elevated in AAD.	(97)
SPP1, VEGFA, CCL20, GDF15, CXCL5, IGFBP3, CXCL14, HMOX1, CA9, CCL14	mRNA microarrays	CA9, CXCL5, GDF15, VEGFA, CCL20, HMOX1 and SPP1 were positively correlated with CD14 (corresponding to monocytes); CA9, CXCL5, GDF15 and VEGFA were positively correlated with CD68 (corresponding to macrophages).	(99)
CDK1, CDC20, CCNB2, CCNB1, MAD2L1, AURKA, C3AR1, NCAPG, CXCL12, ASPM	Microarray technology, MCODE, cytoHubba analyses	Inhibition of CDK1 reduces the proliferation and migration of VSMCs and protects blood vessels.	(98, 100)
RPS9, RPS18, RSRC1, DNAJC3, HBS1L, PRKCA, NCAM1, ITGB3, FTSJ3	GWAS, TWAS	Gene set enrichment analysis suggested that these hub genes mainly involved in mRNA catabolic process, focal adhesion and chemotaxis.	(101)
MRTF-A	Microarray technology	Plays a role in pro-inflammatory and pro-apoptotic processes and promotes AAD.	(102)
SLC20A1, GINS2, CNN1, FAM198B, MAD2L2, UBE2T, FKBP11, SLMAP, CCDC34, GALK1	Microarray technology, WGCNA	It has the strongest positive correlation with AAD	(103)
JAK2, PDGFA, TGFB1, VEGFA, TIMP3, TIMP4, SERPINE1	Microarray technology	Regulation of inflammatory response, growth factor activity and extracellular matrix	(104, 105)
ANGPT2	Microarray technology	ANGPT2 is significantly increased in the aortic intima of AAD patients	(107)
CXCL1, CXCL5, HTR7, SNAP25, EMX2, NCAM1, IGFBP2	RNA sequencing	Differentially expressed in mesenchymal stem cells, regulates the development of AAD	(109)
Legumain	Microarray technology	As an endogenous integrin αvβ3 modulator, Legumain promotes the development of TAD	(110)
ACTA2, IL6, CTGF, BGN, ITGA8, THBS1, CDH5	scRNA-seq	Among them, ITGA8, THBS1 and IL6 were enriched in four related signaling pathways, implying that these genes play a role in promoting metabolism, growth and angiogenesis in VSMC	(111)
ITGA9	Whole gene transcription microarrays	This gene is downregulated in AAD patients and is involved in regulating the phenotypic transition of VSMC from a contractile to a synthetic phenotype.	(114)

### 3.3 Proteomics and the discovery of AAD biomarkers

In proteomics research, some proteins that possess high abundance on their own are easy to detect but lack diagnostic specificity for diseases. To improve the fight against AAD in biomedical disciplines, it is crucial to apply quantitative protein technologies based on mass spectrometry and microarrays to identify and measure some low-abundance proteins that change throughout disease development (119). Future solutions to this technical issue are also anticipated, including multiplexing and targeted proteomics (120) combined with multidimensional and orthogonal separation techniques (121) to improve chromatographic resolution.

Looking around the cell, the extracellular matrix (ECM) is an insoluble structural component that makes up the matrix in the mesenchymal and epithelial capillaries and is mostly composed of collagen, elastin, proteoglycans and glycoproteins. Studies have demonstrated that ECM can impact biological processes such as cell differentiation, proliferation, adhesion, morphogenesis and phenotypic expression. An iTRAQ-based TAD proteomics study screened 36 differentially expressed proteins and validated them using western blotting for fibrillin-1, emilin-1, decorin, protein DJ-1 and histone H4. The results showed that the expression of protein DJ-1 and histone H4 was increased, while the expression of fibrillin-1, emilin-1 and decorin was decreased in TAD patients compared to controls. The results of differential proteomics screening and biological functional analysis also suggested that the major protein interaction networks involved in TAD are inflammation-interleukin 6 (IL-6) signaling, protein hydrolysis-extracellular matrix (ECM) remodeling, and cell adhesion-cell matrix interaction. These networks may be important in the pathogenesis of TAD when combined with the TGF-β signaling pathway (122). Also, by comparing aneurysmal tissue at the ascending aortic site in patients with and without Marfan syndrome (MFS), we demonstrated that microfibrillar-associated glycoprotein 4 (MFAP4) was present in high amounts in the aortic vessel wall of patients with MFS, and by 68 months of follow-up showed that type B entrapment occurred in 5 of the patients with high plasma MFAP4. At the same time, high plasma MFAP4 levels have been shown to be associated with low descending aortic dilatability, whereas the relationship between aortic dilatability in the thoracic descending aorta and type B entrapment has been previously studied (123). In addition, medial degeneration of thoracic aortic aneurysm and dissection (TAAD) is characterized by proteoglycan buildup, which is particularly damaging to the homeostasis of smooth muscle cells and predisposes to type A dissection. Aortic dissection/rupture is linked in Fbn1mgR/mgR mice to accumulation of aggregated proteoglycans and multifunctional proteoglycans in the ascending TAAD, which occurs through increased synthesis and/or impaired protein hydrolysis turnover (124). By using proteomics techniques, nine proteins, including Lumican, FGL1, PI16, MMP9, FBN1, MMP2, VWF, MMRN1, and PF4, that are linked to the pathophysiology of AAD, were also identified. Serum levels of these proteins were found to be considerably higher in AAD patients (125). Among these, Lumican, a tiny leucine-rich proteoglycan, is an essential part of the aorta wall’s extracellular matrix and is crucial for cell division, migration, and differentiation as well as tissue healing. With a diagnostic sensitivity of 73.33% and a specificity of 98.33% for AAD, serum levels of Lumican were shown to be considerably higher in individuals with AAD in another investigation, making it a promising novel biomarker (126, 127). While the Lumican reflects aortic wall damage and repair of ECM proteins, the increased D-dimer indicates excessive fibrinolysis after AAD. The excellent specificity of Lumican for AAD and the excellent sensitivity of D-dimer can be coupled in the diagnosis of AAD, producing a better combined diagnosis with a sensitivity and specificity of 88.33% and 95% simultaneously (128). It was further demonstrated that the sensitivity and specificity could be adjusted to 79.49% and 98.46%, respectively, by combining ANGPTL8, hs-CRP, and D-dimer (126). ACAN proteoglycan was regarded as a trustworthy possible biomarker for ATAAD in plasma samples, with a sensitivity of 97% and specificity of 81% for the diagnosis of AAD, and is anticipated to be paired with additional markers (129). In the future, it is expected to be paired with additional biomarkers for combination diagnosis.

The maintenance of vascular homeostasis depends on the normal contractile function of vascular smooth muscle cells, which is closely related to cell-matrix adhesion. Cell adhesion makes up 27% of the primary biological functions of the proteins that differ in AAD, indicating that matrix remodeling and cell adhesion are significant pathophysiological mechanisms in AAD. The integrin family functions as cell adhesion molecules that mediate cell-extracellular matrix attachment. As biomarkers for the diagnosis of AAD, they include reduced expression of integrin α-3 (ITGA-3), integrin α-5 (ITGA-5) (22), and high expression of integrin α-IIb (ITGA2B), integrin α-M (ITGAM), integrin β-2 (ITGB2), and integrin β-3 (ITGB3) (130). Meanwhile, MMP-9, a key enzyme involved in the breakdown of extracellular matrix and the migration of inflammatory cells, was discovered to be enhanced by integrins in immortalized keratin-forming cells and its inhibitors were able to reduce the incidence of AAD by 40% (131). In the first 48 h after the onset of AAD, cytoskeletal proteins such vinculin have been discovered to sustain high levels, and they may play a part in early identification (132). Other cytoskeletal proteins like cofilin and LIMK are less expressed in thoracic aortic dissection than in healthy controls, which may contribute to the weakening of the medial tissue of the thoracic aorta, increasing the diameter of the aorta and raising the risk of AAD development (133).

Similar to the enrichment findings from transcriptomic analysis, the majority of the differentially expressed proteins for proteins outside of cells showed functions primarily involved in cell migration and proliferation, inflammatory cell activation, cell contraction, oxidative stress, and muscle organ development (130). Extracellular superoxide dismutase, an enzyme involved in oxidative stress, was one of them. Its expression was more than 50% lower in AAD patient samples than in controls, and there was a rise in lipid peroxidation, both of which could point to the onset of AAD (134). Additionally, it has been discovered that many lipid-related proteins, including the carboxypeptidase N catalytic chain proteins (CPNs), complement component proteins, serum amyloid A protein (SAA), and complement component proteins, are differentially expressed in AAD (135). Circulating levels of LDL cholesterol and very low density lipoprotein (VLDL) small particles are also strongly linked to AAD (136).

Additionally, interesting biomarkers to distinguish TAD from emergency patients with significant chest pain included ITGA2, COL2A1, and MIF (137). Interestingly, a comparison of ascending aortic wall specimens from AAD, aneurysms and normal controls by differential in-gel electrophoresis (DIGE) analysis revealed that antitrypsin (A1AT) was found to be reduced in protein amounts in aortic tissue from aortic dissection compared to healthy aorta, but not in aneurysms (138). A1AT was, however, found to be considerably higher in aortic tissue samples from patients with thoracic aortic dissection and hypertension in another iTRAQ-based proteomics investigation (122). For the biological role performed by A1AT in AAD, further research and validation of this expression difference are still needed (Table 3).

**TABLE 3:** Non-exhaustive list of biomarkers for AAD elucidated by proteomic-based techniques.

Biomarker	Technology	Valuable considerations	References
Fibrillin-1, emilin-1, decorin, protein DJ-1 and histone H4	iTRAQ technique	Their differential expression in TAD was identified by protein blotting.	(122)
MFAP4	MS	High plasma MFAP4 levels and more diverse N-glycosylation are associated with a higher incidence of type B aortic coarctation	(123)
Lumican, FGL1, PI16, MMP9, FBN1, MMP2, VWF, MMRN1, PF4	iTRAQ technique	Serum levels of FGL1, PI16 and MMP9 were significantly elevated in AAD patients.	(125)
Lumican	iTRAQ technique	The combined detection of D-dimer and Lumican has better diagnostic value for AAD.	(127)
ACAN	MS	ACAN protein concentration is enhanced in plasma of patients with acute type A aortic dissection.	(129)
ITGA-3, ITGA-5	Amine-reactive tandem mass tag (TMT) labeling, MS	the IOD of ITGA-3 and ITGA-5 in AAD patients was significantly lower than that in healthy donors.	(22)
ITGA2B, ITGAM, ITGB2, ITGB3	MS, 4D-LFQ	These proteins were significantly upregulated in the ascending aorta tissue of AAD patients.	(130)
Vinculin	label-free proteomics approach	Vinculin were found to maintain high levels within 48 h before the onset of AAD.	(132)
Cofilin, LIMK	previous proteomic research	The protein expression of cofilin and LIMK was significantly decreased in thoracic aortic dissection tissue compared with normal control.	(133)
Extracellular superoxide dismutase	MS	Its expression in AAD patient samples is more than 50% lower than that in the control group.	(134)
SAAs, complement component proteins, CPNs	iTRAQ technique	B2-GP1, CPN1, F9, LBP, SAA1, and SAA2, were validated by ELISA.	(135)
FGF6, FGF9, HGF, BCL2L1, VEGFA, ECM1, SPOCK3, IL1b, LDL-C, VLDL-C	GWAS, pQTL	Circulating levels of all these proteins are associated with AAD.	(136)
ITGA2, MIF, COL2A1	label-free quantification proteomics method	It can discriminating TAD from emergency patients with severe chest pain.	(137)
A1AT	iTRAQ technique	One study said it was decreased in AAD, while another study showed it was increased.	(122, 138)

### 3.4 Metabolomics and the discovery of AAD biomarkers

With the development of high performance liquid chromatography-mass spectrometry (HPLC-MS) technology, it is expanding from the field of drug metabolite analysis to the study of biological endogenous metabolites (139), allowing us to gradually study small molecule biomarkers. Peripheral blood metabolite analysis can also be utilized to help in the diagnosis of AAD because AAD changes the metabolome in the peripheral blood. Although peripheral blood testing is a rapid and less invasive technique, there is a lack of validated peripheral blood markers for AAD, especially small molecule metabolite markers (140). Large molecule proteins such smooth muscle myosin heavy chain (smMHC), creatine kinase-BB (CK-BB), D-dimer, MMPs, and elastin are the majority of the existing peripheral blood biomarkers of AAD (141, 142). Thus, it has become a research goal to discover more novel small molecule metabolic biomarkers of AAD.

Most of the entry points for the study of metabolomics were derived from the insights brought by transcriptomics and proteomics. We have discovered from earlier research that macrophages are crucial to the emergence of AAD. And because succinate triggers inflammatory changes in macrophages, Cui et al. found elevated plasma succinate concentrations in AAD patients compared to healthy controls, acute myocardial infarction (AMI) patients, and pulmonary embolism (PE) patients, leading to the idea that plasma succinate concentrations could distinguish AAD from the other three groups and the idea that succinate concentrations are regulated by p38α-CREB-OGDH axis in macrophages (143). Moreover, when macrophages undergo metabolic reprogramming, it leads to the accumulation of ferredoxin salts, which induces the activation of macrophage hypoxia-inducible factor 1-α (HIF-1α), which in turn exacerbates the development of AAD by increasing depolymerization and metallopeptidase structural domain 17 (ADAM17) triggering vascular inflammation, extracellular matrix degradation and elastic plate breakage (144). We also discovered, using multi-omics data in TAD, that elevated ceramide levels may be caused by accelerated ceramide *ab initio* production pathways in macrophages, with C18-ceramide, a key sphingolipid metabolite, being significantly different in TAD patients. Exogenous C18-ceramide is involved in the process of aortic injury by promoting smooth muscle cell contractile protein degradation, macrophage inflammation and MMP9 expression and exacerbating extracellular matrix degradation via the NLRP3-caspase 1 pathway (145, 146). Interestingly, another study found that sphingolipids (including sphingosine, phytosphingosine, sphingomyelin, and ceramide) were significantly reduced in the Stanford A AAD group but not in the Stanford B AAD group, making sphingolipids promising as potential biomarkers to differentiate Stanford A from Stanford B aortic dissection (147). Based on this study, the distinction between type A and type B dissection from a biochemical perspective is a promising finding. Also, on the basis of the theory that the descending segment of the thoracoabdominal aorta originated from the mesoderm in embryonic-based studies, whereas the aortic root and ascending aorta originated from the neural ridge, and that the different embryonic origins led to different gene expression and behavior of vascular smooth muscle cells in various regions of the aorta (88, 148). It also suggests that in the future we can pay more attention to whether the anatomical barrier of the aorta can be considered as a biological/metabolic/protein barrier and thus discover more of the different pathophysiological bases that may exist for type A and type B dissection. And the most variable lipid in AAD, lysophosphatidylcholine (LPC), is reduced in each subtype of the disease and is located at the center of the network linked to lipid changes (149). In recent years, lipidomics has emerged in multi-omics studies of AAD, and more and more biomarkers are beginning to be identified. In addition to the sphingolipids and LPC already mentioned, significant changes in lipid and polar metabolites were discovered in AAD patients by untargeted metabolomic analysis. This was followed by changes in the phosphatidylcholine metabolic pathways observed with targeted metabolomics, which further revealed significantly higher trimethylamine N-oxide (TMAO) levels and lower carnitine, choline, and betaine levels in AAD (150).

Overall, the tryptophan, histidine, glycerophospholipids, ether lipids, choline metabolism (140), and galactose metabolic pathways (151) are largely affected by the metabolites differently expressed in AAD. Among them, AFMK, glycerophosphatidylcholine and ergot sulfur were more than 50-fold elevated in peripheral blood of AAD patients compared to the healthy population (140). With reduced plasma glutamate levels and increased plasma phenylalanine levels in patients with AAD, metabolomics analysis of plasma amino acid profiles also revealed differences in amino acid levels between acute and chronic aortic dissection (152), which may point to a potential role for amino acid levels in the acute pathogenesis of aortic dissection.

In order to reverse the lethal effects of AAD, it is crucial to apply metabolomics techniques to the study of AAD pathogenesis and the identification of specific biomarkers, especially to more precisely and acutely identify changes in the levels of some transient metabolites, which, like the butterfly effect, may reveal a number of significant cascading responses that have not been focused on (Table 4).

**TABLE 4:** Non-exhaustive list of biomarkers for AAD elucidated by metabolomics-based techniques.

Biomarker	Technology	Valuable considerations	References
Succinate	MS	Plasma succinate concentrations were elevated in AAD patients compared with healthy controls, acute myocardial infarction (AMI) patients, and pulmonary embolism (PE) patients.	(143)
C18-ceramide	UPLC-MS/MS	It is significantly increased in patients with TAD but not significantly different in patients with TAA.	(145)
Sphingolipids(including sphingosine, phytosphingosine, and ceramides)	UPLC-MS	They are significantly reduced in the Stanford type A AAD group but not in the Stanford type B AAD group.	(147)
Lysophosphatidylcholine(LPC)	HPLC-MS	Decreased in different types of AAD.	(149)
TMAO, carnitine, choline and betaine	HPLC	The level of TMAO was significantly increased, while the levels of carnitine, choline and betaine were decreased.	(150)
AFMK, glycerophosphocholine, ergothioneine	HPLC-MS	Those levels in AD patient peripheral sera that were more than 50 times higher than in normal human peripheral sera.	(140)
Histidine, Glycine, Serine, Citrate, Ornithine, Hydroxyproline, Proline, Creatine, GABA, Glutamic Acid, Cysteine, Phenylalanine	LC-MS/MS	Pairwise comparison of differences in plasma amino acid levels between acute dissection, chronic dissection, and coronary heart disease.	(152)

## 4 Integration strategies for turning multiple “omics” into “multi-omics”

From each of the omics levels, gene networks associated with the AAD disease can be built using studies from the four primary fields of genomics, transcriptomics, proteomics, and metabolomics. Network level overlap can then be computed. A common study strategy now involves using methods and ideas from systems biology and bioinformatics to find pathways that are enriched, identify Hub genes, and validate them (101, 153). Although separate omics approaches have made a significant contribution to our understanding of AAD, we have yet to identify integrated signaling pathways and networks. The problem of low translation rate in clinical practice continues, and the heterogeneity in sample extraction and data collecting also contributes to a significant gap between clinical and basic research. To better improve the predictive power of basic research for clinical practice, we have gradually focused our attention on more advanced data processing. The use of web-based integrated techniques is growing in popularity quickly, with advantages that go much beyond the simple association of various unfiltered datasets or differentially enriched molecules from several layers. Such integrated approaches (inclusive methods) provide insights not available from any single layer of omics data by exploring causal links between histological candidates and disease conditions/pathways using advanced analysis.

Here, we should focus on the longitudinal integration strategy (154), which is to examine multiple omics variables on the same sample. Denoising, downscaling, and normalizing the dataset beforehand can make it more standard and practical for us, and the data will be more accurate and rigorous. However, it has been a significant problem to combine and interpret numerous noisy and high-dimensional data into a single biological model, which has been a considerable challenge. Prior to analyzing multi-omics data, this necessitates not only performing dimensionality reduction operations based on feature selection or feature extraction on the dataset (155), but also having a solid grasp of the fundamentals of data integration and visualization techniques to make sure the right techniques are applied for the relevant dataset. We must take the matching of multi-omics data into account while selecting a data integration method. For single-cell sequencing, whether measurements are made on the same cells is particularly important for integrated analysis of the data (156). Also, the selection of the dataset is highly related to the main objectives of the analysis and the analysis method that the researcher envisages before collecting the data. If a satisfactory number of samples and depth of coverage cannot be achieved simultaneously, the choice of dataset should be based on the analysis objectives. If the aim is to identify differentially expressed genes between cases and controls, the power provided by more samples is usually preferable to the higher accuracy provided by a higher sequencing depth. However, if the main purpose of the analysis is to identify novel transcripts, or to examine the expression of specific alleles, the deeper the coverage, the better. On the other hand, for the selection of model species, the advantages of using animal models include reproducibility, controllability of environmental factors, availability of relevant tissues, accurate phenotypes, an almost unlimited number of exact biological replicates, and the ability to follow hypotheses experimentally. However, animal models also have limitations. Many gene-specific models are limited to one genetic background, mouse models may not encapsulate the human biology of complex diseases, and some manifestations of human disease may be difficult to test in mouse models. We can help us validate the biological relevance of the models themselves in advance by comparing the histological data between human and animal models, which requires actually specific analysis and selection based on the disease the investigator is studying (2).

In order to analyze complicated data, machine learning (ML) models are frequently utilized. These models can be broadly categorized into supervised learning and unsupervised learning. Unsupervised learning for cluster analysis learning is one of them and is based on data similarity. Five multi-omics integration techniques—JIVE, MCCA, MCIA, MFA, and SNF—representing multivariate, tandem-based, and transformation-based approaches have been chosen to compare their capacity to combine more than two omics data in an unsupervised manner (157), providing us with a benchmark for choosing future multi-omics research techniques in the area of AAD. Deep learning (DL), a branch of machine learning, is an unsupervised learning technique based on model structural variations that enables computers to carry out an automated learning process. Due to their ability to capture non-linear and hierarchical features as well as their predictive accuracy, DL algorithms are among the most promising methods in multi-omics data processing (158). There are also studies that classify integration strategies into five categories: early integration, hybrid integration, intermediate strategies, late integration and hierarchical integration (159). They integrate datasets in different ways at different stages of data processing, and each has its own advantages and disadvantages, requiring us to make specific decisions for different datasets and research purposes. The objectives we always work toward include, however, limiting the amount of data loss and noise while lowering dataset heterogeneity, conserving the properties of unique histological datasets, and recording interactions between various histologies.

In recent years, numerous innovative and distinctive computational modeling frameworks and R programming packages (160, 161) have been created, and academics frequently choose integrated tools or platforms that mix interactive visuals with integrated analytical workflows (28). Examples include the DeepProg methodological model (162), which can explicitly model patient survival as a goal and predict the survival risk of new patients, and the DIABLO methodological model (163), which allows for module-based analysis and cross-sectional study design. It will be more convenient for non-data analysis professions like clinical researchers and will promote interdisciplinary development and collaboration.

## 5 Current shortcomings and future prospects

It is encouraging to see how quickly systems biology technologies are developing, and the idea of using them to treat AAD is become more and more appealing. The development of multi-omics has been aided by the big data era, and applying multi-omics technology to biomedicine is both an opportunity and a challenge. The identification of biomarkers using multi-omics data can aid in stratifying patients, improving prevention, early diagnosis, and prediction, monitoring progression, interpreting patterns and endophenotypes, and creating individualized treatment plans (158, 164).

However, the current state of AAD research demonstrates that multi-omics integration for biomarker exploration is still notably lacking. The only studies available have focused on the combined analysis of genomics and transcriptomics (101). For example, based on the fact that the increased risk of AAD rupture is mostly associated with aortic enlargement and aneurysm development, some scholars screened some potential gene targets based on the integration of genome-wide association studies (GWAS), transcriptome-wide association studies (TWAS), and rare variant analysis, and analyzed their associated cell types in combination with single-cell RAN sequencing, and identified a number of potential targets including VSMCs, fibroblasts, three different types of endothelial cells, macrophage and lymphocyte populations, and examined the precise gene expression patterns in each cell type (117). Another study combined differentially expressed genes (DEGs) of AAD and differentially methylated m6A genes from other datasets, obtained differentially expressed m6A-related genes by gene crossover results, and analyzed the relationship between these genes and immune cell infiltration. This study also explored in greater detail epigenetic mechanisms through DNA/chromatin and histone modifications, and the association with cell that brings new insights into the potential influence of externa. However, as noted in numerous studies, gene expression patterns are frequently utilized to research disease states, despite the fact that proteins are the primary functional components causing the disease. At the same time, the advent of metabolomics and macrogenomics, which integrate the impacts of food intake, utilization, and flow, represents an extra complexity of disease (165). It follows that our future focus and effort in the field of AAD research will be integrated studies combining proteomics, metabolomics, and other histological concepts. We anticipate finding more drug-targeting protein families in the future thanks to multi-omics integration approaches, which will allow us to investigate the molecular targets and potential pharmacological action mechanisms on AAD (166). The future therapy pathway for AAD will also include a critical stage involving the screening of biomarkers for acute pharmacological inhibitors, which may also serve as a model for the construction of drug resistance profiles. At the same time, we should also actively apply and integrate some of the newly emerged omics concepts in the future. For example, using the concept of radiomics to digitize image information, merge it with sequencing information and establish correlation, it may be possible to propose new molecular biology-based pathological typing for AAD based on large sample multi-omics sequencing (167).

Each of these new concepts will significantly advance our understanding of the biological networks and linkages in AAD. As a result of their strong interconnections, clusters of similar pathways naturally occur, giving us the opportunity to gather the key biological themes in each stage or layer of the disease. In particular, the resulting pathway networks could offer a global view of the various processes involved in the disease (168). This would be especially important for a disease like AAD, which has multiple sites of pathogenesis and is very dynamic. We will be able to learn more about the pathogenic mechanisms of AAD by enhancing routes and building interaction networks. This indicates that in the future, employing multi-omics technologies for their bioinformatic analysis and validation, we can more frequently draw new inspiration and insights into AAD research from other diseases with similar pathophysiology or risk factors for its development. For instance, using microarray and bioinformatics analysis, we discovered that ALDH2 deficiency downregulates miR-31-5p, which further modifies cardiac myosin mRNA levels and attenuates abnormal VSMC phenotypic transition to achieve the impact of avoiding the formation of AAD (169). However, inactivating mutations in ALDH2 (called ALDH2*2 or rs671 mutants) are risk factors for the development and poor prognosis of atherosclerotic cardiovascular disease. By using RNA-seq, proteomics, and immunoprecipitation experiments, we later discovered and verified Rac2 as its downstream target, demonstrating the crucial function of the ALDH2-Rac2 axis in mediating cytokinesis during atherosclerosis (170). As a result, many genes are present in the body as a “double-edged sword,” and even in diseases that are linked to specific mutations, they can have opposing effects. To create more dependable, scientific, and accountable healthcare decisions, it is crucial to evaluate them more thoroughly using multi-omics technology.

In addition to the above mentioned shortcomings in the application of multi-omics technologies, there is also much room for development and enhancement of the omics technologies themselves. One of them is the advent of single-cell analysis, which promises a deeper exploration in the field of proteins and allows for the quantification of more than 1000 proteins from a single mammalian cell, has the potential to advance biomedical research and provide a new degree of granularity in our understanding of biological systems. Future proteomics technologies, which have the ability to do away with the necessity for antibodies and open up paths for minimally biased research into single cell protein expression, are equally promising (171). And when it comes to operational standards, the species specificity should be carefully taken into account throughout the whole multi-omics study process (172, 173), particularly during screening and validation, as failure to do so could result in an inadequately enriched analysis of various datasets. Orthometry, machine learning, and signaling networks are being used in computational methods being actively developed to increase the translatability of omics datasets between preclinical models and humans (174). To produce findings that can be applied, technical histological expertise must be paired with fundamental biological knowledge. In the future, we anticipate being able to standardize and rigorize studies to the fullest extent possible in order to produce data with a higher signal-to-noise ratio and a more comprehensive integrated analysis. Meanwhile, massive data sharing systems like MarkerDB (58) will also accelerate this field’s development. We anticipate that by addressing the aforementioned concerns, precision medicine will be able to move beyond the traditional approach of signs and symptoms and create more individualized treatment plans for patients based on diagnoses that incorporate clinical, lifestyle, genetic, and other biomarkers.

## 6 Conclusion

Using the idea of multi-omics integration and gathering data from all levels, we have employed and attempted to develop more advanced systems biology methodologies to investigate the underlying molecular mechanisms of AAD from surface to deeper levels. To lower the missed diagnosis rate of AAD and enhance illness prognosis, we will look into new medication treatment targets with the aim of identifying better bio-diagnostic indicators. Our initial goal in attempting to find solutions to all issues will always be to cure the condition and, ideally, in the future, improve patient quality of life.

## Author contributions

XH conducted the literature review, drafted the manuscript, and prepared the figures. SC and BJ revised the manuscript. All authors have substantially contributed to the article and approved the submitted version.

## References

[1] Zhou M Varol A Efferth T. Multi-omics approaches to improve malaria therapy. *Pharmacol Res.* (2021) 167:105570. 10.1016/j.phrs.2021.105570 33766628

[2] Hasin Y Seldin M Lusis A. Multi-omics approaches to disease. *Genome Biol.* (2017) 18:83. 10.1186/s13059-017-1215-1 28476144 PMC5418815

[3] Wilkins MR Sanchez JC Gooley AA Appel RD Humphery-Smith I Hochstrasser DF Progress with proteome projects: why all proteins expressed by a genome should be identified and how to do it. *Biotechnol Genet Eng Rev.* (1996) 13:19–50. 10.1080/02648725.1996.10647923 8948108

[4] Chakraborty S Hosen MI Ahmed M Shekhar HU. Onco-multi-OMICS approach: a new frontier in cancer research. *Biomed Res Int.* (2018) 2018:9836256. 10.1155/2018/9836256 30402498 PMC6192166

[5] Karczewski KJ Snyder MP. Integrative omics for health and disease. *Nat Rev Genet.* (2018) 19:299–310. 10.1038/nrg.2018.4 29479082 PMC5990367

[6] Yang TL Shen H Liu A Dong SS Zhang L Deng FY A road map for understanding molecular and genetic determinants of osteoporosis. *Nat Rev Endocrinol.* (2020) 16:91–103. 10.1038/s41574-019-0282-7 31792439 PMC6980376

[7] Fukushima A Kanaya S Nishida K. Integrated network analysis and effective tools in plant systems biology. *Front Plant Sci.* (2014) 5:598. 10.3389/fpls.2014.00598 25408696 PMC4219401

[8] Dwivedi S Purohit P Misra R Lingeswaran M Vishnoi JR Pareek P Single cell omics of breast cancer: an update on characterization and diagnosis. *Indian J Clin Biochem.* (2019) 34:3–18. 10.1007/s12291-019-0811-0 30728668 PMC6346617

[9] Liu G Dong C Liu L. Integrated multiple “-omics” data reveal subtypes of hepatocellular carcinoma. *PLoS One.* (2016) 11:e0165457. 10.1371/journal.pone.0165457 27806083 PMC5091875

[10] Xiao Y Bi M Guo H Li M. Multi-omics approaches for biomarker discovery in early ovarian cancer diagnosis. *EBioMedicine.* (2022) 79:104001. 10.1016/j.ebiom.2022.104001 35439677 PMC9035645

[11] Ghosh D Bernstein JA Khurana Hershey GK Rothenberg ME Mersha TB. Leveraging multilayered “omics” data for atopic dermatitis: a road map to precision medicine. *Front Immunol.* (2018) 9:2727. 10.3389/fimmu.2018.02727 30631320 PMC6315155

[12] Hou X Qu H Zhang S Qi X Hakonarson H Xia Q The multi-omics architecture of juvenile idiopathic arthritis. *Cells.* (2020) 9:2301. 10.3390/cells9102301 33076506 PMC7602566

[13] Bernardes JP Mishra N Tran F Bahmer T Best L Blase JI Longitudinal multi-omics analyses identify responses of megakaryocytes, erythroid cells, and plasmablasts as hallmarks of severe COVID-19. *Immunity.* (2020) 53:1296–314.e9. 10.1016/j.immuni.2020.11.017 33296687 PMC7689306

[14] Doran S Arif M Lam S Bayraktar A Turkez H Uhlen M Multi-omics approaches for revealing the complexity of cardiovascular disease. *Brief Bioinform.* (2021) 22:bbab061. 10.1093/bib/bbab061 33725119 PMC8425417

[15] Olivier M Asmis R Hawkins GA Howard TD Cox LA. The need for multi-omics biomarker signatures in precision medicine. *Int J Mol Sci.* (2019) 20:4781. 10.3390/ijms20194781 31561483 PMC6801754

[16] Aboyans V Boukhris M. Dissecting the epidemiology of aortic dissection. *Eur Heart J Acute Cardiovasc Care.* (2021) 10:710–1. 10.1093/ehjacc/zuab065 34389858

[17] Zeng T Shi L Ji Q Shi Y Huang Y Liu Y Cytokines in aortic dissection. *Clin Chim Acta* (2018) 486:177–82. 10.1016/j.cca.2018.08.005 30086263

[18] Tchana-Sato V Sakalihasan N Defraigne JO. [Aortic dissection]. *Rev Med Liege.* (2018) 73:290–5. 29926568

[19] Gawinecka J Schönrath F von Eckardstein A. Acute aortic dissection: pathogenesis, risk factors and diagnosis. *Swiss Med Wkly.* (2017) 147:w14489. 10.4414/smw.2017.14489 28871571

[20] Manea MM Dragos D Antonescu F Sirbu AG Tiron AT Dobri AM Aortic dissection: an easily missed diagnosis when pain doesn’t hold the stage. *Am J Case Rep.* (2019) 20:1788–92. 10.12659/AJCR.917179 31786581 PMC6910182

[21] Silaschi M Byrne J Wendler O. Aortic dissection: medical, interventional and surgical management. *Heart.* (2017) 103:78–87. 10.1136/heartjnl-2015-308284 27733536

[22] Xing L Xue Y Yang Y Wu P Wong CCL Wang H Proteomic analysis identification of integrin alpha 3 and integrin alpha 5 as novel biomarkers in pathogenesis of acute aortic dissection. *Biomed Res Int.* (2020) 2020:1068402. 10.1155/2020/1068402 32851057 PMC7441460

[23] Moore AG Eagle KA Bruckman D Moon BS Malouf JF Fattori R Choice of computed tomography, transesophageal echocardiography, magnetic resonance imaging, and aortography in acute aortic dissection: international registry of acute aortic dissection (IRAD). *Am J Cardiol.* (2002) 89:1235–8. 10.1016/s0002-9149(02)02316-0 12008187

[24] Kristensen VN Lingjærde OC Russnes HG Vollan HK Frigessi A ørresen-Dale ALB. Principles and methods of integrative genomic analyses in cancer. *Nat Rev Cancer.* (2014) 14:299–313. 10.1038/nrc3721 24759209

[25] Kopczynski D Coman C Zahedi RP Lorenz K Sickmann A Ahrends R. Multi-OMICS: a critical technical perspective on integrative lipidomics approaches. *Biochim Biophys Acta Mol Cell Biol Lipids.* (2017) 1862:808–11. 10.1016/j.bbalip.2017.02.003 28193460

[26] Visscher PM Brown MA McCarthy MI Yang J. Five years of GWAS discovery. *Am J Hum Genet.* (2012) 90:7–24. 10.1016/j.ajhg.2011.11.029 22243964 PMC3257326

[27] Xing C Huang J Hsu YH DeStefano AL Heard-Costa NL Wolf PA Evaluation of power of the illumina humanomni5M-4v1 beadchip to detect risk variants for human complex diseases. *Eur J Hum Genet.* (2016) 24:1029–34. 10.1038/ejhg.2015.244 26577045 PMC5070895

[28] Chu X Zhang B Koeken V Gupta MK Li Y. Multi-omics approaches in immunological research. *Front Immunol.* (2021) 12:668045. 10.3389/fimmu.2021.668045 34177908 PMC8226116

[29] McMahon A Lewis E Buniello A Cerezo M Hall P Sollis E Sequencing-based genome-wide association studies reporting standards. *Cell Genom.* (2021) 1:100005. 10.1016/j.xgen.2021.100005 34870259 PMC8637874

[30] Milewicz DM Regalado ES Shendure J Nickerson DA Guo DC. Successes and challenges of using whole exome sequencing to identify novel genes underlying an inherited predisposition for thoracic aortic aneurysms and acute aortic dissections. *Trends Cardiovasc Med.* (2014) 24:53–60. 10.1016/j.tcm.2013.06.004 23953976 PMC3917689

[31] Mardis ER. Next-generation DNA sequencing methods. *Annu Rev Genomics Hum Genet.* (2008) 9:387–402. 18576944 10.1146/annurev.genom.9.081307.164359

[32] Heather JM Chain B. The sequence of sequencers: the history of sequencing DNA. *Genomics.* (2016) 107:1–8. 10.1016/j.ygeno.2015.11.003 26554401 PMC4727787

[33] Wang Y Zhao Y Bollas A Wang Y Au KF. Nanopore sequencing technology, bioinformatics and applications. *Nat Biotechnol.* (2021) 39:1348–65. 10.1038/s41587-021-01108-x 34750572 PMC8988251

[34] Jiang Z Zhou X Li R Michal JJ Zhang S Dodson MV Whole transcriptome analysis with sequencing: methods, challenges and potential solutions. *Cell Mol Life Sci.* (2015) 72:3425–39. 26018601 10.1007/s00018-015-1934-y PMC6233721

[35] Soon WW Hariharan M Snyder MP. High-throughput sequencing for biology and medicine. *Mol Syst Biol.* (2013) 9:640. 10.1038/msb.2012.61 PMC3564260 23340846

[36] Longo SK Guo MG Ji AL Khavari PA. Integrating single-cell and spatial transcriptomics to elucidate intercellular tissue dynamics. *Nat Rev Genet.* (2021) 22:627–44. 10.1038/s41576-021-00370-8 34145435 PMC9888017

[37] van der Wijst MGP Brugge H de Vries DH Deelen P Swertz MA Franke L. Single-cell RNA sequencing identifies celltype-specific cis-eQTLs and co-expression QTLs. *Nat Genet.* (2018) 50:493–7. 10.1038/s41588-018-0089-9 29610479 PMC5905669

[38] Ziegenhain C Vieth B Parekh S Reinius B Guillaumet-Adkins A Smets M Comparative analysis of single-cell RNA sequencing methods. *Mol Cell.* (2017) 65:631–43.e4. 28212749 10.1016/j.molcel.2017.01.023

[39] Ståhl PL Salmén F Vickovic S Lundmark A Navarro JF Magnusson J Visualization and analysis of gene expression in tissue sections by spatial transcriptomics. *Science.* (2016) 353:78–82. 27365449 10.1126/science.aaf2403

[40] Rao A Barkley D França GS Yanai I. Exploring tissue architecture using spatial transcriptomics. *Nature.* (2021) 596:211–20. 10.1038/s41586-021-03634-9 34381231 PMC8475179

[41] Baccin C Al-Sabah J Velten L Helbling PM Grünschläger F Hernández-Malmierca P Combined single-cell and spatial transcriptomics reveal the molecular, cellular and spatial bone marrow niche organization. *Nat Cell Biol.* (2020) 22:38–48. 10.1038/s41556-019-0439-6 31871321 PMC7610809

[42] Domon B Aebersold R. Mass spectrometry and protein analysis. *Science.* (2006) 312:212–7. 10.1016/s1570-0232(02)00125-3 16614208

[43] Smith LM Kelleher NL. Proteoform: a single term describing protein complexity. *Nat Methods.* (2013) 10:186–7. 10.1038/nmeth.2369 23443629 PMC4114032

[44] Zhang Z Wu S Stenoien DL Paša-Tolić L. High-throughput proteomics. *Annu Rev Anal Chem (Palo Alto Calif).* (2014) 7:427–54. 10.1146/annurev-anchem-071213-020216 25014346

[45] Butler GS Dean RA Morrison CJ Overall CM. Identification of cellular MMP substrates using quantitative proteomics: isotope-coded affinity tags (ICAT) and isobaric tags for relative and absolute quantification (iTRAQ). *Methods Mol Biol.* (2010) 622:451–70. 10.1007/978-1-60327-299-5_26 20135298

[46] Chen X Wei S Ji Y Guo X Yang F. Quantitative proteomics using SILAC: principles, applications, and developments. *Proteomics.* (2015) 15:3175–92. 26097186 10.1002/pmic.201500108

[47] Shi Y. A glimpse of structural biology through X-ray crystallography. *Cell.* (2014) 159:995–1014. 25416941 10.1016/j.cell.2014.10.051

[48] Breindel L Burz DS Shekhtman A. Interaction proteomics by using in-cell NMR spectroscopy. *J Proteomics.* (2019) 191:202–11. 29427760 10.1016/j.jprot.2018.02.006 PMC6082733

[49] Aslam B Basit M Nisar MA Khurshid M Rasool MH. Proteomics: technologies and their applications. *J Chromatogr Sci.* (2017) 55:182–96. 28087761 10.1093/chromsci/bmw167

[50] Altelaar AF Munoz J Heck AJ. Next-generation proteomics: towards an integrative view of proteome dynamics. *Nat Rev Genet.* (2013) 14:35–48. 10.1038/nrg3356 23207911

[51] Seger C Salzmann L. After another decade: LC-MS/MS became routine in clinical diagnostics. *Clin Biochem.* (2020) 82:2–11. 10.1016/j.clinbiochem.2020.03.004 32188572

[52] Oliver SG Winson MK Kell DB Baganz F. Systematic functional analysis of the yeast genome. *Trends Biotechnol.* (1998) 16:373–8. 10.1016/S0167-7799(98)01214-1 9744112

[53] Gertsman I Barshop BA. Promises and pitfalls of untargeted metabolomics. *J Inherit Metab Dis.* (2018) 41:355–66. 29536203 10.1007/s10545-017-0130-7 PMC5960440

[54] Bauermeister A Mannochio-Russo H Costa-Lotufo LV Jarmusch AK Dorrestein PC. Mass spectrometry-based metabolomics in microbiome investigations. *Nat Rev Microbiol.* (2022) 20:143–60. 34552265 10.1038/s41579-021-00621-9 PMC9578303

[55] Chong J Soufan O Li C Caraus I Li S Bourque G MetaboAnalyst 4.0: towards more transparent and integrative metabolomics analysis. *Nucleic Acids Res.* (2018) 46:W486–94. 10.1093/nar/gky310 29762782 PMC6030889

[56] Vasan RS. Biomarkers of cardiovascular disease: molecular basis and practical considerations. *Circulation.* (2006) 113:2335–62. 16702488 10.1161/CIRCULATIONAHA.104.482570

[57] Rifai N Gillette MA Carr SA. Protein biomarker discovery and validation: the long and uncertain path to clinical utility. *Nat Biotechnol.* (2006) 24:971–83. 10.1038/nbt1235 16900146

[58] Wishart DS Bartok B Oler E Liang KYH Budinski Z Berjanskii M MarkerDB: an online database of molecular biomarkers. *Nucleic Acids Res.* (2021) 49:D1259–67. 10.1093/nar/gkaa1067 33245771 PMC7778954

[59] Zhou Z Liu Y Zhu X Tang X Wang Y Wang J Exaggerated Autophagy in stanford type A aortic dissection: a transcriptome pilot analysis of human ascending aortic tissues. *Genes.* (2020) 11:1187. 10.3390/genes11101187 33066131 PMC7650806

[60] Sen I Erben YM Franco-Mesa C DeMartino RR. Epidemiology of aortic dissection. *Semin Vasc Surg.* (2021) 34:10–7. 33757630 10.1053/j.semvascsurg.2021.02.003

[61] Gago-Díaz M Ramos-Luis E Zoppis S Zorio E Molina P Braza-Boïls A Postmortem genetic testing should be recommended in sudden cardiac death cases due to thoracic aortic dissection. *Int J Legal Med.* (2017) 131:1211–9. 10.1007/s00414-017-1583-9 28391405

[62] Renard M Francis C Ghosh R Scott AF Witmer PD Adès LC Clinical validity of genes for heritable thoracic aortic aneurysm and dissection. *J Am Coll Cardiol.* (2018) 72:605–15. 30071989 10.1016/j.jacc.2018.04.089 PMC6378369

[63] Grond-Ginsbach C Pjontek R Aksay SS Hyhlik-Dürr A Böckler D Gross-Weissmann ML. Spontaneous arterial dissection: phenotype and molecular pathogenesis. *Cell Mol Life Sci.* (2010) 67:1799–815. 10.1007/s00018-010-0276-z 20155481 PMC11115591

[64] Gago-Díaz M Blanco-Verea A Teixidó G Huguet F Gut M Laurie S PRKG1 and genetic diagnosis of early-onset thoracic aortic disease. *Eur J Clin Invest.* (2016) 46:787–94. 10.1111/eci.12662 27442293

[65] Guo DC Regalado E Casteel DE Santos-Cortez RL Gong L Kim JJ Recurrent gain-of-function mutation in PRKG1 causes thoracic aortic aneurysms and acute aortic dissections. *Am J Hum Genet.* (2013) 93:398–404. 10.1016/j.ajhg.2013.06.019 23910461 PMC3738837

[66] Van Laer L Dietz H Loeys B. Loeys-Dietz syndrome. *Adv Exp Med Biol.* (2014) 802:95–105. 10.1007/978-94-007-7893-1_7 24443023

[67] Regalado ES Guo DC Villamizar C Avidan N Gilchrist D McGillivray B Exome sequencing identifies SMAD3 mutations as a cause of familial thoracic aortic aneurysm and dissection with intracranial and other arterial aneurysms. *Circ Res.* (2011) 109:680–6. 10.1161/CIRCRESAHA.111.248161 21778426 PMC4115811

[68] Blinc A Maver A Rudolf G Tasič J Pretnar Oblak J Berden P Clinical exome sequencing as a novel tool for diagnosing Loeys-dietz syndrome type 3. *Eur J Vasc Endovasc Surg.* (2015) 50:816–21. 10.1016/j.ejvs.2015.08.003 26409702

[69] Engström K Vánky F Rehnberg M Trinks C Jonasson J Green A Novel SMAD3 p.Arg386Thr genetic variant co-segregating with thoracic aortic aneurysm and dissection. *Mol Genet Genomic Med.* (2020) 8:e1089. 10.1002/mgg3.1089 32022471 PMC7196476

[70] Cannaerts E Kempers M Maugeri A Marcelis C Gardeitchik T Richer J Novel pathogenic SMAD2 variants in five families with arterial aneurysm and dissection: further delineation of the phenotype. *J Med Genet.* (2019) 56:220–7. 10.1136/jmedgenet-2018-105304 29967133

[71] Duan XY Guo DC Regalado ES Shen H Coselli JS Estrera AL SMAD4 rare variants in individuals and families with thoracic aortic aneurysms and dissections. *Eur J Hum Genet.* (2019) 27:1054–60. 10.1038/s41431-019-0357-x 30809044 PMC6777456

[72] Wang Y Huang HY Bian GL Yu YS Ye WX Hua F of SMAD4 enhances thoracic aortic aneurysm and dissection risk through promoting smooth muscle cell apoptosis and proteoglycan degradation. *EBioMedicine.* (2017) 21:197–205. 10.1016/j.ebiom.2017.06.022 28666732 PMC5514432

[73] Xu S Li L Fu Y Wang X Sun H Wang J Increased frequency of FBN1 frameshift and nonsense mutations in Marfan syndrome patients with aortic dissection. *Mol Genet Genomic Med.* (2020) 8:e1041. 10.1002/mgg3.1041 31830381 PMC6978253

[74] Erhart P Gieldon L Ante M Körfer D Strom T Grond-Ginsbach C Acute Stanford type B aortic dissection-who benefits from genetic testing? *J Thorac Dis.* (2020) 12:6806–12. 10.21037/jtd-20-2421 33282382 PMC7711383

[75] Li J Yang L Diao Y Zhou L Xin Y Jiang L Genetic testing and clinical relevance of patients with thoracic aortic aneurysm and dissection in northwestern China. *Mol Genet Genomic Med.* (2021) 9:e1800. 10.1002/mgg3.1800 34498425 PMC8580079

[76] Chen ZR Bao MH Wang XY Yang YM Huang B Han ZL Genetic variants in Chinese patients with sporadic Stanford type A aortic dissection. *J Thorac Dis.* (2021) 13:4008–22. 10.21037/jtd-20-2758 34422331 PMC8339749

[77] Guo DC Regalado ES Gong L Duan X Santos-Cortez RL Arnaud P LOX mutations predispose to thoracic aortic aneurysms and dissections. *Circ Res.* (2016) 118:928–34. 26838787 10.1161/CIRCRESAHA.115.307130 PMC4839295

[78] Lee VS Halabi CM Hoffman EP Carmichael N Leshchiner I Lian CG Loss of function mutation in LOX causes thoracic aortic aneurysm and dissection in humans. *Proc Natl Acad Sci U.S.A.* (2016) 113:8759–64. 10.1073/pnas.1601442113 27432961 PMC4978273

[79] Guo DC Regalado ES Pinard A Chen J Lee K Rigelsky C LTBP3 pathogenic variants predispose individuals to thoracic aortic aneurysms and dissections. *Am J Hum Genet.* (2018) 102:706–12. 10.1016/j.ajhg.2018.03.002 29625025 PMC5985335

[80] Yang H Zhu G Zhou W Luo M Zhang Y Zhang Y A systematic study of mosaicism in heritable thoracic aortic aneurysm and dissection. *Genomics.* (2022) 114:196–201. 10.1016/j.ygeno.2021.12.002 34921932

[81] Prakash S Kuang SQ Regalado E Guo D Milewicz D. Recurrent rare genomic copy number variants and bicuspid aortic valve are enriched in early onset thoracic aortic aneurysms and dissections. *PLoS One.* (2016) 11:e0153543. 10.1371/journal.pone.0153543 27092555 PMC4836726

[82] Overwater E Marsili L Baars MJH Baas AF van deBeek I Dulfer E Results of next-generation sequencing gene panel diagnostics including copy-number variation analysis in 810 patients suspected of heritable thoracic aortic disorders. *Hum Mutat.* (2018) 39:1173–92. 10.1002/humu.23565 29907982 PMC6175145

[83] Bergeron SE Wedemeyer EW Lee R Wen KK McKane M Pierick AR Allele-specific effects of thoracic aortic aneurysm and dissection alpha-smooth muscle actin mutations on actin function. *J Biol Chem.* (2011) 286:11356–69. 10.1074/jbc.M110.203174 21288906 PMC3064192

[84] Liu P Zhang J Du D Zhang D Jin Z Qiu W Altered DNA methylation pattern reveals epigenetic regulation of Hox genes in thoracic aortic dissection and serves as a biomarker in disease diagnosis. *Clin Epigenet.* (2021) 13:124. 10.1186/s13148-021-01110-9 34103071 PMC8186232

[85] Maredia A Guzzardi D Aleinati M Iqbal F Khaira A Madhu A Aorta-specific DNA methylation patterns in cell-free DNA from patients with bicuspid aortic valve-associated aortopathy. *Clin Epigenet.* (2021) 13:147. 10.1186/s13148-021-01137-y 34321094 PMC8320174

[86] Li N Lin H Zhou H Zheng D Xu G Shi H Efficient detection of differentially methylated regions in the genome of patients with thoracic aortic dissection and association with MMP2 hypermethylation. *Exp Ther Med.* (2020) 20:1073–81. 10.3892/etm.2020.8753 32765660 PMC7388572

[87] Pan S Lai H Shen Y Breeze C Beck S Hong T DNA methylome analysis reveals distinct epigenetic patterns of ascending aortic dissection and bicuspid aortic valve. *Cardiovasc Res.* (2017) 113:692–704. 10.1093/cvr/cvx050 28444195

[88] Fletcher AJ Syed MBJ Aitman TJ Newby DE Walker NL. Inherited thoracic aortic disease: new insights and translational targets. *Circulation.* (2020) 141:1570–87. 10.1161/CIRCULATIONAHA.119.043756 32392100 PMC7217141

[89] Zhang X Yang Z Li X Liu X Wang X Qiu T Bioinformatics analysis reveals cell cycle-related gene upregulation in ascending aortic tissues from murine models. *Front Genet.* (2022) 13:823769. 10.3389/fgene.2022.823769 35356426 PMC8959095

[90] Li Z Zhou C Tan L Chen P Cao Y Li C Variants of genes encoding collagens and matrix metalloproteinase system increased the risk of aortic dissection. *Sci China Life Sci.* (2017) 60:57–65. 10.1007/s11427-016-0333-3 27975164

[91] Chen Y Sun Y Li Z Li C Xiao L Dai J Identificationof COL3A1 variants associated with sporadic thoracic aortic dissection: a case-control study. *Front Med.* (2021) 15:438–47. 10.1007/s11684-020-0826-1 34047934

[92] Guo DC Grove ML Prakash SK Eriksson P Hostetler EM LeMaire SA Genetic variants in LRP1 and ULK4 are associated with acute aortic dissections. *Am J Hum Genet.* (2016) 99:762–9. 10.1016/j.ajhg.2016.06.034 27569546 PMC5011062

[93] Chai T Tian M Yang X Qiu Z Lin X Chen L. Association of circulating cathepsin B levels with blood pressure and aortic dilation. *Front Cardiovasc Med.* (2022) 9:762468. 10.3389/fcvm.2022.762468 35425820 PMC9001941

[94] Wolford BN Hornsby WE Guo D Zhou W Lin M Farhat L Clinical implications of identifying pathogenic variants in individuals with thoracic aortic dissection. *Circ Genom Precis Med.* (2019) 12:e002476. 10.1161/CIRCGEN.118.002476 PMC6582991 31211624

[95] Chang Y Yuan Q Jiang P Sun L Ma Y Ma X. Association of gene polymorphisms in MYH11 and TGF-β signaling with the susceptibility and clinical outcomes of DeBakey type III aortic dissection. *Mamm Genome.* (2022) 33:555–63. 10.1007/s00335-021-09929-6 34729648

[96] Tcheandjieu C Xiao K Tejeda H Lynch JA Ruotsalainen S Bellomo T High heritability of ascending aortic diameter and trans-ancestry prediction of thoracic aortic disease. *Nat Genet.* (2022) 54:772–82. 10.1038/s41588-022-01070-7 35637384 PMC13102105

[97] Wang W Wang T Wang Y Piao H Li B Zhu Z Integration of Gene expression profile data to verify hub genes of patients with Stanford A aortic dissection. *Biomed Res Int.* (2019) 2019:3629751. 10.1155/2019/3629751 31380418 PMC6662449

[98] Wang W Liu Q Wang Y Piao H Li B Zhu Z Verification of hub genes in the expression profile of aortic dissection. *PLoS One.* (2019) 14:e0224922. 10.1371/journal.pone.0224922 31751374 PMC6872142

[99] Gao H Sun X Liu Y Liang S Zhang B Wang L Analysis of Hub genes and the mechanism of immune infiltration in Stanford type A aortic dissection. *Front Cardiovasc Med.* (2021) 8:680065. 10.3389/fcvm.2021.680065 34277731 PMC8284479

[100] Jiang T Si L. Identification of the molecular mechanisms associated with acute type A aortic dissection through bioinformatics methods. *Braz J Med Biol Res.* (2019) 52:e8950. 10.1590/1414-431X20198950 31721906 PMC6853077

[101] Zhang Y Li L Ma L. Integrative analysis of transcriptome-wide association study and mRNA expression profile identified candidate genes and pathways associated with aortic aneurysm and dissection. *Gene.* (2022) 808:145993. 10.1016/j.gene.2021.145993 34626721

[102] Ito S Hashimoto Y Majima R Nakao E Aoki H Nishihara M MRTF-A promotes angiotensin II-induced inflammatory response and aortic dissection in mice. *PLoS One.* (2020) 15:e0229888. 10.1371/journal.pone.0229888 32208430 PMC7092993

[103] Wang T He X Liu X Liu Y Zhang W Huang Q Weighted Gene Co-expression network analysis identifies FKBP11 as a key regulator in acute aortic dissection through a NF-kB dependent pathway. *Front Physiol.* (2017) 8:1010. 10.3389/fphys.2017.01010 29255427 PMC5723018

[104] Kimura N Futamura K Arakawa M Okada N Emrich F Okamura H Gene expression profiling of acute type A aortic dissection combined with in vitro assessment. *Eur J Cardiothorac Surg.* (2017) 52:810–7. 10.1093/ejcts/ezx095 28402522

[105] Pan S Wu D Teschendorff AE Hong T Wang L Qian M JAK2-centered interactome hotspot identified by an integrative network algorithm in acute Stanford type A aortic dissection. *PLoS One.* (2014) 9:e89406. 10.1371/journal.pone.0089406 24586754 PMC3933461

[106] Weis-Müller BT Modlich O Drobinskaya I Unay D Huber R Bojar H Gene expression in acute Stanford type A dissection: a comparative microarray study. *J Transl Med.* (2006) 4:29. 10.1186/1479-5876-4-29 PMC1557406 16824202

[107] Huang B Tian L Chen Z Zhang L Su W Lu T Angiopoietin 2 as a novel potential biomarker for acute aortic dissection. *Front Cardiovasc Med.* (2021) 8:743519. 10.3389/fcvm.2021.743519 35004874 PMC8733161

[108] Liu Y Zou L Tang H Li J Liu H Jiang X Single-cell sequencing of immune cells in human aortic dissection tissue provides insights into immune cell heterogeneity. *Front Cardiovasc Med.* (2022) 9:791875. 10.3389/fcvm.2022.791875 PMC9008490 35433892

[109] Yang J Zou S Liao M Qu L. Transcriptome sequencing revealed candidate genes relevant to mesenchymal stem cells’ role in aortic dissection patients. *Mol Med Rep.* (2018) 17:273–83. 10.3892/mmr.2017.7851 29115411 PMC5780137

[110] Pan L Bai P Weng X Liu J Chen Y Chen S Legumain is an endogenous modulator of integrin αvβ3 triggering vascular degeneration, dissection, and rupture. *Circulation.* (2022) 145:659–74. 10.1161/CIRCULATIONAHA.121.056640 35100526

[111] Xu C Liu X Fang X Yu L Lau HC Li D Single-cell RNA sequencing reveals smooth muscle cells heterogeneity in experimental aortic dissection. *Front Genet.* (2022) 13:836593. 10.3389/fgene.2022.836593 36035191 PMC9403608

[112] Chen Y Zhang T Yao F Gao X Li D Fu S Dysregulation of interaction between LOX(high) fibroblast and smooth muscle cells contributes to the pathogenesis of aortic dissection. *Theranostics.* (2022) 12:910–28. 10.7150/thno.66059 34976220 PMC8692905

[113] Shao Y Li G Huang S Li Z Qiao B Chen D Effects of extracellular matrix softening on vascular smooth muscle cell dysfunction. *Cardiovasc Toxicol.* (2020) 20:548–56. 10.1007/s12012-020-09580-8 32500384

[114] Huang B Niu Y Chen Z Yang Y Wang X. Integrin α9 is involved in the pathopoiesis of acute aortic dissection via mediating phenotype switch of vascular smooth muscle cell. *Biochem Biophys Res Commun.* (2020) 533:519–25. 10.1016/j.bbrc.2020.08.095 32981677

[115] Fu X He X Yang Y Jiang S Wang S Peng X Identification of transfer RNA-derived fragments and their potential roles in aortic dissection. *Genomics.* (2021) 113:3039–49. 10.1016/j.ygeno.2021.06.039 34214628

[116] Zhou Z Liu Y Gao S Zhou M Qi F Ding N Excessive DNA damage mediates ECM degradation via the RBBP8/NOTCH1 pathway in sporadic aortic dissection. *Biochim Biophys Acta Mol Basis Dis.* (2022) 1868:166303. 10.1016/j.bbadis.2021.166303 34780912

[117] Pirruccello JP Chaffin MD Chou EL Fleming SJ Lin H Nekoui M Deep learning enables genetic analysis of the human thoracic aorta. *Nat Genet.* (2022) 54:40–51. 10.1038/s41588-021-00962-4 34837083 PMC8758523

[118] Li YH Cao Y Liu F Zhao Q Adi D Huo Q Visualization and analysis of gene expression in Stanford type A aortic dissection tissue section by spatial transcriptomics. *Front Genet.* (2021) 12:698124. 10.3389/fgene.2021.698124 34262602 PMC8275070

[119] Wu J Wang W Chen Z Xu F Zheng Y. Proteomics applications in biomarker discovery and pathogenesis for abdominal aortic aneurysm. *Expert Rev Proteomics.* (2021) 18:305–14. 33840337 10.1080/14789450.2021.1916473

[120] Pappireddi N Martin L Wühr M. A review on quantitative multiplexed proteomics. *Chembiochem.* (2019) 20:1210–24. 30609196 10.1002/cbic.201800650 PMC6520187

[121] Antberg L Cifani P Sandin M Levander F James P. Critical comparison of multidimensional separation methods for increasing protein expression coverage. *J Proteome Res.* (2012) 11:2644–52. 10.1021/pr201257y 22449141

[122] Zhang K Pan X Zheng J Xu D Zhang J Sun L. Comparative tissue proteomics analysis of thoracic aortic dissection with hypertension using the iTRAQ technique. *Eur J Cardiothorac Surg.* (2015) 47:431–8. 10.1093/ejcts/ezu171 24760388

[123] Yin 殷晓科 X Wanga S Fellows AL Barallobre-Barreiro J Lu R Davaapil H Glycoproteomic analysis of the aortic extracellular matrix in marfan patients. *Arterioscler Thromb Vasc Biol.* (2019) 39:1859–73. 10.1161/ATVBAHA.118.312175 31315432 PMC6727943

[124] Cikach FS Koch CD Mead TJ Galatioto J Willard BB Emerton KB Massive aggrecan and versican accumulation in thoracic aortic aneurysm and dissection. *JCI Insight.* (2018) 3:e97167. 10.1172/jci.insight.97167 29515038 PMC5922288

[125] Deng T Liu Y Gael A Fu X Deng X Liu Y Study on proteomics-based aortic dissection molecular markers using iTRAQ combined with label free techniques. *Front Physiol.* (2022) 13:862732. 10.3389/fphys.2022.862732 35910577 PMC9335284

[126] Yang Y Jiao X Li L Hu C Zhang X Pan L Increased circulating angiopoietin-like protein 8 levels are associated with thoracic aortic dissection and higher inflammatory conditions. *Cardiovasc Drugs Ther.* (2020) 34:65–77. 32034642 10.1007/s10557-019-06924-7 PMC7093348

[127] Gu G Cheng W Yao C Yin J Tong C Rao A Quantitative proteomics analysis by isobaric tags for relative and absolute quantitation identified Lumican as a potential marker for acute aortic dissection. *J Biomed Biotechnol.* (2011) 2011:920763. 10.1155/2011/920763 PMC3250623 22228989

[128] Xiao Z Xue Y Yao C Gu G Zhang Y Zhang J Acute aortic dissection biomarkers identified using isobaric tags for relative and absolute quantitation. *Biomed Res Int.* (2016) 2016:6421451. 10.1155/2016/6421451 PMC4925974 27403433

[129] König KC Lahm H Dreßen M Doppler SA Eichhorn S Beck N Aggrecan: a new biomarker for acute type A aortic dissection. *Sci Rep.* (2021) 11:10371. 10.1038/s41598-021-89653-y PMC8121825 33990642

[130] Wang MM Wang BZ Adi D Shao MH Zhang D Lu CF [Analysis on tissue-related biomarkers in patients with acute aortic dissection]. *Zhonghua Xin Xue Guan Bing Za Zhi.* (2021) 49:1108–16. 10.3760/cma.j.cn112148-20210929-00839 34775721

[131] Li X Liu D Zhao L Wang L Li Y Cho K Targeted depletion of monocyte/macrophage suppresses aortic dissection with the spatial regulation of MMP-9 in the aorta. *Life Sci.* (2020) 254:116927. 10.1016/j.lfs.2019.116927 31672577

[132] Wang HQ Yang H Tang Q Gong YC Fu YH Wan F Identification of vinculin as a potential diagnostic biomarker for acute aortic dissection using label-free proteomics. *Biomed Res Int.* (2020) 2020:7806409. 10.1155/2020/7806409 32766314 PMC7388002

[133] Tian L Liao MF Zhang L Lu QS Jing ZP. A study of the expression and interaction of Destrin, cofilin, and LIMK in Debakey I type thoracic aortic dissection tissue. *Scand J Clin Lab Invest.* (2010) 70:523–8. 10.3109/00365513.2010.521572 20873970

[134] Liao M Liu Z Bao J Zhao Z Hu J Feng X A proteomic study of the aortic media in human thoracic aortic dissection: implication for oxidative stress. *J Thorac Cardiovasc Surg.* (2008) 136:65–72, 72.e1–3. 10.1016/j.jtcvs.2007.11.017 18603055

[135] Cheng N Wang H Zhang W Wang H Jin X Ma X Comparative proteomic investigation of plasma reveals novel potential biomarker groups for acute aortic dissection. *Dis Markers.* (2020) 2020:4785068. 10.1155/2020/4785068 32256857 PMC7106916

[136] Chai T Tian M Yang X Qiu Z Lin X Chen L. Genome-wide identification of associations of circulating molecules with spontaneous coronary artery dissection and aortic aneurysm and dissection. *Front Cardiovasc Med.* (2022) 9:874912. 10.3389/fcvm.2022.874912 35571188 PMC9091499

[137] Qiu P Yang M Pu H Hou J Chen X Wu Z Potential clinical value of biomarker-guided emergency triage for thoracic aortic dissection. *Front Cardiovasc Med.* (2021) 8:777327. 10.3389/fcvm.2021.777327 35096998 PMC8790093

[138] Schachner T Golderer G Sarg B Lindner HH Bonaros N Mikuz G The amounts of alpha 1 antitrypsin protein are reduced in the vascular wall of the acutely dissected human ascending aorta. *Eur J Cardiothorac Surg.* (2010) 37:684–90. 10.1016/j.ejcts.2009.07.025 19709897

[139] Lu X Zhao X Bai C Zhao C Lu G Xu G LC-MS-based metabonomics analysis. *J Chromatogr B Analyt Technol Biomed Life Sci.* (2008) 866:64–76. 10.1016/j.jchromb.2007.10.022 17983864

[140] Ren Y Tang Q Liu W Tang Y Zhu R Li B. Serum biomarker identification by mass spectrometry in acute aortic dissection. *Cell Physiol Biochem.* (2017) 44:2147–57. 29241182 10.1159/000485954

[141] Suzuki T Katoh H Watanabe M Kurabayashi M Hiramori K Hori S Novel biochemical diagnostic method for aortic dissection. Results of a prospective study using an immunoassay of smooth muscle myosin heavy chain. *Circulation.* (1996) 93:1244–9. 10.1161/01.cir.93.6.1244 8653847

[142] Apostolakis E Akinosoglou K. What’s new in the biochemical diagnosis of acute aortic dissection: problems and perspectives. *Med Sci Monit.* (2007) 13:Ra154–8. 17660735

[143] Cui H Chen Y Li K Zhan R Zhao M Xu Y Untargeted metabolomics identifies succinate as a biomarker and therapeutic target in aortic aneurysm and dissection. *Eur Heart J.* (2021) 42:4373–85. 10.1093/eurheartj/ehab605 34534287 PMC11506060

[144] Lian G Li X Zhang L Zhang Y Sun L Zhang X Macrophage metabolic reprogramming aggravates aortic dissection through the HIF1α-ADAM17 pathway (✰). *EBioMedicine.* (2019) 49:291–304. 10.1016/j.ebiom.2019.09.041 31640947 PMC6945268

[145] Yang H Yang F Luo M Chen Q Liu X Zhang Y Metabolomic profile reveals that ceramide metabolic disturbance plays an important role in thoracic aortic dissection. *Front Cardiovasc Med.* (2022) 9:826861. 10.3389/fcvm.2022.826861 35211530 PMC8861291

[146] Doppler C Arnhard K Dumfarth J Heinz K Messner B Stern C Metabolomic profiling of ascending thoracic aortic aneurysms and dissections - Implications for pathophysiology and biomarker discovery. *PLoS One.* (2017) 12:e0176727. 10.1371/journal.pone.0176727 28467501 PMC5415060

[147] Zhou X Wang R Zhang T Liu F Zhang W Wang G Identification of lysophosphatidylcholines and sphingolipids as potential biomarkers for acute aortic dissection via serum metabolomics. *Eur J Vasc Endovasc Surg.* (2019) 57:434–41. 10.1016/j.ejvs.2018.07.004 30087010

[148] Murillo H Lane MJ Punn R Fleischmann D Restrepo CS. Imaging of the aorta: embryology and anatomy. *Semin Ultrasound CT MR.* (2012) 33:169–90. 22624964 10.1053/j.sult.2012.01.013

[149] Huang H Ye G Lai SQ Zou HX Yuan B Wu QC Plasma lipidomics identifies unique lipid signatures and potential biomarkers for patients with aortic dissection. *Front Cardiovasc Med.* (2021) 8:757022. 10.3389/fcvm.2021.757022 34778409 PMC8581228

[150] Zeng Q Rong Y Li D Wu Z He Y Zhang H Identification of serum biomarker in acute aortic dissection by global and targeted metabolomics. *Ann Vasc Surg.* (2020) 68:497–504. 32599111 10.1016/j.avsg.2020.06.026

[151] Zhang K Pan X Zheng J Liu Y Sun L. The metabolic analysis in human aortic tissues of aortic dissection. *J Clin Lab Anal.* (2022) 36:e24623. 10.1002/jcla.24623 PMC9459286 35881684

[152] Wang L Liu S Yang W Yu H Zhang L Ma P Plasma amino acid profile in patients with aortic dissection. *Sci Rep.* (2017) 7:40146. 10.1038/srep40146 PMC5223271 28071727

[153] Yin F Zhang H Guo P Wu Y Zhao X Li F Comprehensive analysis of key m6A modification related genes and immune infiltrates in human aortic dissection. *Front Cardiovasc Med.* (2022) 9:831561. 10.3389/fcvm.2022.831561 35369349 PMC8967178

[154] Zitnik M Nguyen F Wang B Leskovec J Goldenberg A Hoffman MM. Machine learning for integrating data in biology and medicine: principles, practice, and opportunities. *Inf Fusion.* (2019) 50:71–91. 10.1016/j.inffus.2018.09.012 30467459 PMC6242341

[155] Meng C Zeleznik OA Thallinger GG Kuster B Gholami AM Culhane AC. Dimension reduction techniques for the integrative analysis of multi-omics data. *Brief Bioinform.* (2016) 17:628–41. 10.1093/bib/bbv108 26969681 PMC4945831

[156] Miao Z Humphreys BD McMahon AP Kim J. Multi-omics integration in the age of million single-cell data. *Nat Rev Nephrol.* (2021) 17:710–24. 10.1038/s41581-021-00463-x 34417589 PMC9191639

[157] Tini G Marchetti L Priami C Scott-Boyer MP. Multi-omics integration-a comparison of unsupervised clustering methodologies. *Brief Bioinform.* (2019) 20:1269–79. 29272335 10.1093/bib/bbx167

[158] Kang M Ko E Mersha TB. A roadmap for multi-omics data integration using deep learning. *Brief Bioinform.* (2022) 23:bbab454. 10.1093/bib/bbab454 PMC8769688 34791014

[159] Picard M Scott-Boyer MP Bodein A Périn O Droit A. Integration strategies of multi-omics data for machine learning analysis. *Comput Struct Biotechnol J.* (2021) 19:3735–46. 34285775 10.1016/j.csbj.2021.06.030 PMC8258788

[160] Ulfenborg B. Vertical and horizontal integration of multi-omics data with miodin. *BMC Bioinformatics.* (2019) 20:649. 10.1186/s12859-019-3224-4 31823712 PMC6902525

[161] Bodein A Scott-Boyer MP Perin O Lê Cao KA Droit A. timeOmics: an R package for longitudinal multi-omics data integration. *Bioinformatics*. (2021) 38:577–9. 10.1093/bioinformatics/btab664 34554215

[162] Poirion OB Jing Z Chaudhary K Huang S Garmire LX. DeepProg: an ensemble of deep-learning and machine-learning models for prognosis prediction using multi-omics data. *Genome Med.* (2021) 13:112. 10.1186/s13073-021-00930-x PMC8281595 34261540

[163] Singh A Shannon CP Gautier B Rohart F Vacher M Tebbutt SJ DIABLO: an integrative approach for identifying key molecular drivers from multi-omics assays. *Bioinformatics.* (2019) 35:3055–62. 30657866 10.1093/bioinformatics/bty1054 PMC6735831

[164] Reel PS Reel S Pearson E Trucco E Jefferson E. Using machine learning approaches for multi-omics data analysis: a review. *Biotechnol Adv.* (2021) 49:107739. 10.1016/j.biotechadv.2021.107739 33794304

[165] Leon-Mimila P Wang J Huertas-Vazquez A. Relevance of multi-omics studies in cardiovascular diseases. *Front Cardiovasc Med.* (2019) 6:91. 10.3389/fcvm.2019.00091 31380393 PMC6656333

[166] Liang Z Zhang Y Chen Q Hao J Wang H Li Y Analysis of MCM proteins’ role as a potential target of statins in patients with acute type A aortic dissection through bioinformatics. *Genes.* (2021) 12:387. 10.3390/genes12030387 PMC7998850 33803192

[167] Gillies RJ Kinahan PE Hricak H. Radiomics: images are more than pictures. They are data. *Radiology.* (2016) 278:563–77. 26579733 10.1148/radiol.2015151169 PMC4734157

[168] Schlotter F Halu A Goto S Blaser MC Body SC Lee LH Spatiotemporal multi-omics mapping generates a molecular atlas of the aortic valve and reveals networks driving disease. *Circulation.* (2018) 138: 377–93. 29588317 10.1161/CIRCULATIONAHA.117.032291 PMC6160370

[169] Yang K Ren J Li X Wang Z Xue L Cui S Prevention of aortic dissection and aneurysm via an ALDH2-mediated switch in vascular smooth muscle cell phenotype. *Eur Heart J.* (2020) 41:2442–53. 32428930 10.1093/eurheartj/ehaa352

[170] Zhang J Zhao X Guo Y Liu Z Wei S Yuan Q Macrophage ALDH2 (aldehyde dehydrogenase 2) stabilizing Rac2 is required for efferocytosis internalization and reduction of atherosclerosis development. *Arterioscler Thromb Vasc Biol.* (2022) 42:700–16. 35354308 10.1161/ATVBAHA.121.317204 PMC9126264

[171] Vistain LF Tay S. Single-cell proteomics. *Trends Biochem Sci.* (2021) 46:661–72. 10.1016/j.tibs.2021.01.013 33653632 PMC11697639

[172] Shen H Lu S Dong L Xue Y Yao C Tong C hsa-miR-320d and hsa-miR-582, miRNA biomarkers of aortic dissection, regulate apoptosis of vascular smooth muscle cells. *J Cardiovasc Pharmacol.* (2018) 71:275–82. 10.1097/FJC.0000000000000568 29538087

[173] Wineinger NE Patki A Meyers KJ Broeckel U Gu CC Rao DC Genome-wide joint SNP and CNV analysis of aortic root diameter in African Americans: the HyperGEN study. *BMC Med Genomics.* (2011) 4:4. 10.1186/1755-8794-4-4 21223598 PMC3027088

[174] Blaser MC Kraler S Lüscher TF Aikawa E. Multi-omics approaches to define calcific aortic valve disease pathogenesis. *Circ Res.* (2021) 128:1371–97. 10.1161/CIRCRESAHA.120.317979 33914608 PMC8095729

